# Potentiating the cellular targeting and anti-tumor activity of Dp44mT *via* binding to human serum albumin: two saturable mechanisms of Dp44mT uptake by cells

**DOI:** 10.18632/oncotarget.3606

**Published:** 2015-03-15

**Authors:** Angelica M. Merlot, Sumit Sahni, Darius J.R. Lane, Ashleigh M. Fordham, Namfon Pantarat, David E. Hibbs, Vera Richardson, Munikumar R. Doddareddy, Jennifer A. Ong, Michael L.H. Huang, Des R. Richardson, Danuta S. Kalinowski

**Affiliations:** ^1^ Molecular Pharmacology and Pathology Program, Department of Pathology and Bosch Institute, The University of Sydney, Sydney, NSW, Australia; ^2^ Faculty of Pharmacy, The University of Sydney, Sydney, NSW, Australia

**Keywords:** Albumin, Dp44mT, Anti-tumor targeting, Human serum albumin

## Abstract

Di-2-pyridylketone 4,4-dimethyl-3-thiosemicarbazone (Dp44mT) demonstrates potent anti-cancer activity. We previously demonstrated that ^14^C-Dp44mT enters and targets cells through a carrier/receptor-mediated uptake process. Despite structural similarity, 2-benzoylpyridine 4-ethyl-3-thiosemicarbazone (Bp4eT) and pyridoxal isonicotinoyl hydrazone (PIH) enter cells *via* passive diffusion. Considering albumin alters the uptake of many drugs, we examined the effect of human serum albumin (HSA) on the cellular uptake of Dp44mT, Bp4eT and PIH. Chelator-HSA binding studies demonstrated the following order of relative affinity: Bp4eT≈PIH>Dp44mT. Interestingly, HSA decreased Bp4eT and PIH uptake, potentially due to its high affinity for the ligands. In contrast, HSA markedly stimulated Dp44mT uptake by cells, with two saturable uptake mechanisms identified. The first mechanism saturated at 5-10 μM (*B_max_*:1.20±0.04 × 10^7^ molecules/cell; *K_d_*:33±3 μM) and was consistent with a previously identified Dp44mT receptor/carrier. The second mechanism was of lower affinity, but higher capacity (*B_max_*:2.90±0.12 × 10^7^ molecules/cell; *K_d_*:65±6 μM), becoming saturated at 100 μM and was only evident in the presence of HSA. This second saturable Dp44mT uptake process was inhibited by excess HSA and had characteristics suggesting it was mediated by a specific binding site. Significantly, the HSA-mediated increase in the targeting of Dp44mT to cancer cells potentiated apoptosis and could be important for enhancing efficacy.

## INTRODUCTION

Various exogenous compounds, including drugs such as warfarin [1], chlorpromazine [2], digitoxin [2] and ibuprofen [2], as well as endogenous molecules, such as fatty acids [3], steroids [4, 5] and inorganic ions [5, 6], bind extensively to albumin. In general, drug-protein interactions can adversely affect drug delivery by decreasing free drug levels available to traverse the plasma membrane to reach intracellular targets [7-9].

Conversely, albumin has also been demonstrated to aid the uptake and targeting of albumin-bound molecules, including fatty acids [5, 10, 11]. Studies have suggested that, upon binding to the cell membrane, albumin undergoes a conformational change, which subsequently results in the release of albumin-bound fatty acids [5, 11]. The release of the fatty acids in the vicinity of the membrane potentiates the delivery of these molecules to their receptor for cellular uptake [5, 11]. The subsequent reduced affinity of albumin for the cell surface then allows its release from the membrane [5, 11]. This mechanism has also been described for the cellular uptake of other albumin-bound molecules, such as testosterone and tryptophan, demonstrating the biological importance of albumin in the transport and delivery of a variety of ligands [5, 12-14].

Interestingly, albumin has been observed to accumulate within the interstitium of solid tumors [15-17]. This occurs due to the highly permeable tumor vasculature and the insufficient lymphatic drainage present in tumor tissue [15-17]. This characteristic is specifically known as the “enhanced permeability and retention effect” [15-17].

**Figure 1:**
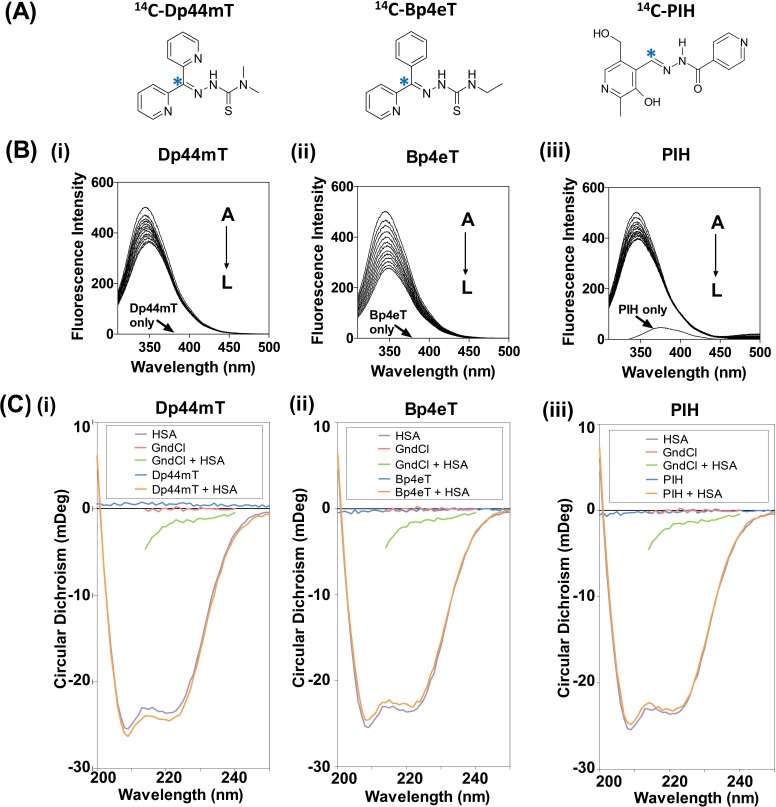
(A): Line drawings of the chemical structures of the iron chelators: di-2-pyridylketone 4,4-dimethyl-3-thiosemicarbazone (Dp44mT), 2-benzoylpyridine 4-ethyl-3-thiosemicarbazone (Bp4eT) and pyridoxal isonicotinoyl hydrazone (PIH) Asterisk (*) indicates position of the ^14^C-label. (B) Fluorescence emission spectrum of HSA (2 μM) excited at 295 nm in the presence of increasing concentrations (A→L; 0-3.67 μM) of: (i) Dp44mT; (ii) Bp4eT; or (iii) PIH in PBS at 37°C/pH 7.4. (C) Circular dichroism of HSA (2 μM) in the presence of: (i) Dp44mT, (ii) Bp4eT or (iii) PIH (10 μM) after a 2 h incubation at 37°C. Results shown are typical of 3 experiments performed.

Thiosemicarbazone ligands are anti-cancer agents that bind metal ions and have shown anti-tumor activity in numerous investigations *in vitro* and *in vivo*, including many clinical trials [18-22]. As part of a specific strategy to generate selective and active anti-tumor agents, the di-2-pyridylketone thiosemicarbazones were developed [18, 19, 23-25]. In particular, the ligand, di-2-pyridylketone 4,4-dimethyl-3-thiosemicarbazone (Dp44mT; Fig. 1A) and its analogs, were shown to have potent *in vitro* and *in vivo* anti-tumor activity [18, 24-26] and to possess marked anti-metastatic efficacy [27-29]. Additionally, the activity of Dp44mT was potentiated in drug-resistant cancer cells [24].

In terms of its mechanism of action, Dp44mT accumulates within lysosomes, where it forms redox-active metal complexes [23, 25, 30] that mediate lysosomal membrane permeabilization to induce apoptosis [31]. Other modes of action include inhibition of the rate-limiting step of DNA synthesis that is catalyzed by ribonucleotide reductase [32] and up-regulation of N-myc downstream regulated gene 1 [33], resulting in inhibition of proliferation and metastasis, respectively [24, 26, 27].

Interestingly, it has been recently demonstrated that Dp44mT binds to a saturable receptor/carrier on a variety of cell-types [34]. Other structurally-related thiosemicarbazones, such as 2-benzoylpyridine 4-ethyl-3-thiosemicarbazone (Bp4eT; Fig. 1A), or aroylhydrazones (e.g., pyridoxal isonicotinoyl hydrazone, PIH; Fig. 1A), entered cells *via* a non-saturable mechanism consistent with passive diffusion [34, 35]. The role of this receptor/carrier in targeting Dp44mT to cancer cells could be important for explaining the marked anti-tumor and anti-metastatic activity, which markedly surpasses other similar agents [18, 24-29].

Considering the increased distribution of albumin in the tumor interstitium and the crucial role of this protein as a drug shuttle [36], it was critical to evaluate the interaction between Dp44mT and albumin. In order to understand the importance of key structural features of Dp44mT in its uptake, studies were performed in comparison to the related ligands, Bp4eT and PIH (Fig. 1A), which possess high and low anti-proliferative activity, respectively [37, 38].

Herein, for the first time, we describe a novel mechanism involved in the cellular uptake and targeting of Dp44mT that is markedly facilitated by human serum albumin (HSA). Intriguingly, this process is distinct from Dp44mT's structurally similar analogs, Bp4eT and PIH, whose cellular uptake was inhibited by HSA. Two saturable mechanisms of Dp44mT uptake by cells were identified. The first uptake mechanism saturated at 5-10 μM, and this observation was consistent with the previously identified Dp44mT receptor/carrier [34]. In contrast, the second mechanism of Dp44mT uptake was a low affinity, high capacity process which saturated at >100 μM and was only evident in the presence of HSA. The enhanced uptake of Dp44mT by HSA was identified in multiple neoplastic cell-types and a normal cell-type. Moreover, the HSA-mediated increase in Dp44mT uptake was specific for this protein and was inhibited by excess HSA. The enhanced cellular targeting of Dp44mT by HSA potentiated the anti-proliferative and apoptotic effects of the agent, facilitating its anti-tumor efficacy.

## RESULTS

### Fluorescence Quenching of HSA by Chelators Indicates Direct Ligand-Binding

Fluorescence spectroscopy was initially used to examine the ability of the ligands to bind HSA (Fig. 1Bi-iii). It is well known that HSA contains a single tryptophan (Trp-214) situated in sub-domain IIA that fluoresces upon excitation at 295 nm [39, 40]. The conformational state of HSA can influence the exposure of this tryptophan residue, and thereby affect tryptophan fluorescence [39].

HSA alone had a pronounced fluorescence maximum at 345 nm (Fig. 1Bi-iii), due to Trp-214 [5]. No minimal intrinsic fluorescence was demonstrated for Dp44mT, Bp4eT, or PBS alone (Fig. 1Bi, ii). In contrast, some intrinsic fluorescence was observed for PIH (Fig. 1Biii). The fluorescence intensity of HSA decreased with increasing concentrations of all the ligands (*i.e.*, A→L; 0-3.67 μM chelator concentrations; see Fig. 1Bi-iii), indicating the interaction of these agents with HSA.

### Circular Dichroism (CD) Spectroscopy Demonstrated No Secondary Conformational Alteration in HSA after Incubation with Dp44mT, Bp4eT or PIH

To determine whether the ligands induce a change in the protein conformation of HSA, changes in protein secondary structure after incubation with Dp44mT, Bp4eT, or PIH, were examined using CD spectroscopy (Fig. 1Ci-iii). The CD spectrum of HSA exhibited two negative peaks at 208 and 222 nm that are characteristic of its predominantly α-helical structure (Fig. 1Ci-iii) [41]. In fact, analysis of the secondary structure revealed 67.73% α-helices (Table 1), which is in agreement with the X-ray crystal structure of HSA [42]. Importantly, no marked alterations in secondary structure were detected after incubation with these ligands (Fig. 1Ci-iii), resulting in similar levels of α-helical secondary structure content (66.86 – 67.84%; Table 1). Interestingly, minimal levels of β-sheet content (5.91%; Table 1) were detected and were not appreciably altered in the presence of the chelators (5.82-6.02%; Table 1). As previously observed [43], the chaotrope and positive control for protein folding, guanidine hydrochloride (GndCl), resulted in the denaturation of HSA (Fig. 1Ci-iii), as demonstrated by a decrease in α-helix content to 1.79%, and the simultaneous increase in β-sheet content to 34.84% (Table 1). Collectively, these results suggest the ligands, Dp44mT, Bp4eT and PIH, do not mediate significant changes in the secondary structure of HSA upon binding.

**Table 1:** The effect of the ligands, Dp44mT, Bp4eT or PIH, or the chaotropic agent, guanidine hydrochloride (GndCl), on α-helix and β-sheet content of HSA

	% α-Helix	% β-Sheets
HSA	67.73%	5.91%
HSA + Dp44mT	67.84%	5.89%
HSA + Bp4eT	66.86%	6.02%
HSA + PIH	67.17%	5.82%
HSA + GndCl*	1.79%	34.84%

* Note: GndCl is used as a positive control to induce alterations in HSA secondary structure.

### ^14^C-Bp4eT and ^14^C-PIH Bind to HSA More Avidly than ^14^C-Dp44mT

**Figure 2:**
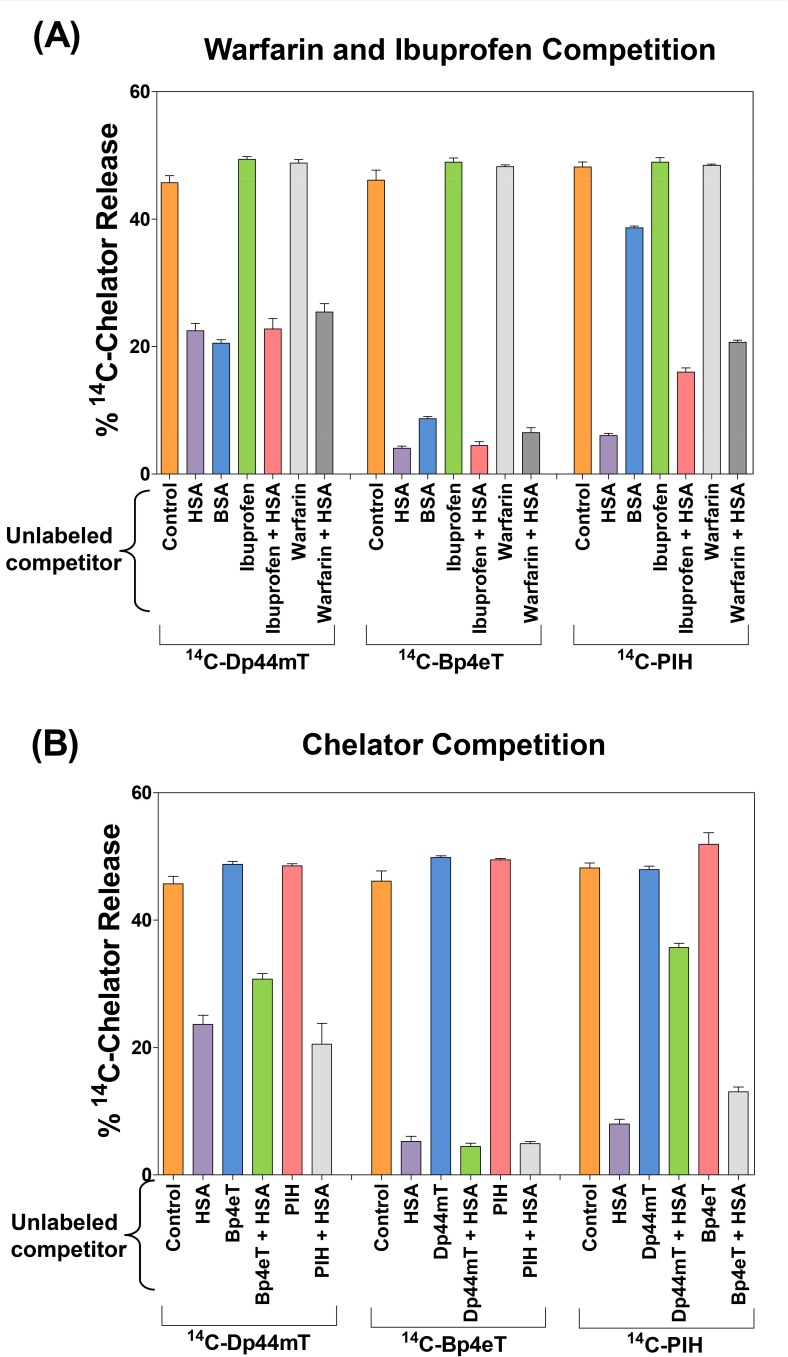
Equilibrium dialysis studies demonstrating the binding of Dp44mT, Bp4eT or PIH to albumin In these studies, HSA or BSA (40 mg/mL) was pre-incubated with the ^14^C-chelators (25 μM) for 2 h/37°C and placed into dialysis sacs and the release of the ^14^C-chelators from the dialysis sac into the dialysate was then examined after a 24 h/4°C equilibration period. For competition studies: (A) HSA (40 mg/mL) was pre-incubated for 2 h/37°C with a 200-fold excess of unlabeled warfarin or ibuprofen (5 mM), or (B) a 20-fold excess of unlabeled Dp44mT, Bp4eT or PIH (0.5 mM). Results are expressed as mean ± S.E.M. of at least 3 experiments.

Further studies were then performed to characterize the binding of the ligands to HSA using the equilibrium dialysis technique [44]. In these experiments, a physiologically-relevant concentration of HSA in the plasma (40 mg/mL; [41, 45]) was pre-incubated with the ^14^C-chelators (25 μM) for 2 h/37°C to duplicate the incubation period used in subsequent experiments examining uptake of the ^14^C-ligands by cells. BSA (40 mg/mL) was chosen as a control protein since it possesses 75.6% sequence identity with HSA [5]. These solutions were then placed into dialysis sacs and the release of the ^14^C-chelators from the sac into the dialysate was then examined after 24 h/4°C to ensure equilibrium (Fig. 2A).

In the absence of protein (*i.e.*, see “control” in Fig. 2A), dialysis of ^14^C-ligands was performed against the buffer-only control and led to the release of ≈ 50% of each of the ^14^C-ligands from the dialysis sac into the dialysate (Fig. 2A). This observation demonstrated that the incubation period was sufficient to establish the equilibrium of these low molecular weight ligands between the dialysis sac and dialysate.

The presence of HSA inside the dialysis sac resulted in a marked and significant (*p*<0.001) decrease in the release of ^14^C-Dp44mT, ^14^C-Bp4eT and ^14^C-PIH into the dialysate to 22.0 ± 1.0%, 4.0 ± 0.3% and 6.0 ± 0.3%, respectively, compared to the relative control (*i.e.*, control sacs without HSA; Fig. 2A). These results demonstrated that the ^14^C-ligands were directly binding to HSA and being retained in the dialysis sac. Clearly, HSA retained the ^14^C-labeled ligands to different extents, with ^14^C-Dp44mT binding significantly (*p*<0.001) less avidly than either ^14^C-Bp4eT or ^14^C-PIH, which bound to HSA with approximately similar avidity (Fig. 2A). These findings indicate the relative binding affinity of the ligands for HSA to be in the following order: ^14^C-Bp4eT ≈^14^C-PIH > ^14^C-Dp44mT (Fig. 2A).

Moreover, BSA (40 mg/mL) also significantly (*p*<0.001) decreased the percentage of ^14^C-Dp44mT, ^14^C-Bp4eT and ^14^C-PIH released from the sac to 21 ± 0.5%, 9 ± 0.3% and 39 ± 0.3%, respectively, compared to control (*i.e.*, control sacs without protein; Fig. 2A). Further, no significant difference (*p*>0.05) in the binding of Dp44mT to either HSA or BSA was evident (Fig. 2A). These data also demonstrate that Bp4eT and PIH bind BSA significantly (*p*<0.001) less avidly relative to HSA.

Considering the HSA-binding described above, we next examined if the ^14^C-ligands (25 μM) bind to the classical drug-binding sites of HSA, namely, Sudlow's site I and/or site II [5]. In these studies, a standard competition protocol was used, whereby HSA (40 mg/mL) was pre-incubated for 2 h/37°C with a 200-fold excess of unlabeled warfarin or ibuprofen (5 mM; Fig. 2A) that bind to Sudlow's sites I or II, respectively [5]. The ^14^C-chelators (25 μM) were then added and the samples further incubated for 2 h/37°C, followed by a 24 h/4°C dialysis period. As a control, in the absence of HSA, equilibrium dialysis of the ^14^C-ligands for 24 h/4°C was also performed in the presence of an excess of unlabeled ibuprofen or warfarin.

Irrespective of the excess warfarin or ibuprofen, the distribution of the ^14^C-ligands reached equilibrium (*i.e.*, the ^14^C-ligand reached ≈50% in both the dialysis sac and dialysate) in the absence of HSA (Fig. 2A). In the presence of ibuprofen or warfarin and HSA, no significant (*p*>0.05) alteration in ^14^C-Dp44mT release from the dialysis sac occurred when compared to ^14^C-Dp44mT and HSA alone (Fig. 2A). These findings suggest that Dp44mT does not compete effectively with warfarin or ibuprofen at Sudlow's site I and II, respectively. Incubating HSA with a molar excess of warfarin, but not ibuprofen, led to a slight, albeit significant (*p*<0.01) increase in ^14^C-Bp4eT release from the dialysis sac when compared to HSA alone (Fig. 2A). This observation suggests that ^14^C-Bp4eT binds to a limited extent to Sudlow's site I, or in the vicinity of this site. More importantly, ^14^C-PIH release from the HSA-containing dialysis sac was markedly and significantly (*p*<0.001) increased in the presence of an excess of warfarin or ibuprofen, relative to the incubation of ^14^C-PIH with HSA alone (Fig. 2A).

Overall, these data suggest: (1) PIH either directly competes with warfarin and ibuprofen for Sudlow's sites I and II, respectively, or that PIH binds HSA at other sites that are allosterically modulated by warfarin- or ibuprofen-binding to HSA; (2) Bp4eT sparingly competes with warfarin at Sudlow's site I only; and (3) Dp44mT does not significantly (*p*>0.05) compete with warfarin or ibuprofen.

### Competition Experiments Reveal HSA has Common and/or Interacting Binding Sites for the Ligands

Competition experiments using equilibrium dialysis were also used to evaluate if the three ^14^C-ligands became bound to a common, or different, site on HSA. Binding of ^14^C-Dp44mT, ^14^C-Bp4eT, or ^14^C-PIH (25 μM) to HSA was examined in competition with a 20-fold excess (0.5 mM) of the relevant unlabeled competitor ligand, namely Dp44mT, Bp4eT or PIH, which was pre-incubated with HSA for 2 h/37°C prior to adding the ^14^C-ligand (Fig. 2B). The unlabeled chelator could only be used at a 20-fold excess relative to the ^14^C-ligand due to their limited solubility. The solutions were then placed into dialysis sacs and the release of ^14^C-ligands from the dialysis sac into the dialysate examined after 24 h/4°C.

Incubation of HSA with an excess of unlabeled Bp4eT induced a slight, but significantly (*p*<0.05) increased release of ^14^C-Dp44mT from the dialysis sac relative to that found in the absence of this unlabeled ligand (Fig. 2B). This observation suggested competition between unlabeled Bp4eT and ^14^C-Dp44mT for a common HSA-binding region (Fig. 2B). However, no significant (*p*>0.05) change in the release of the ^14^C-label into the dialysate was evident when an excess of unlabeled PIH was incubated with ^14^C-Dp44mT and HSA relative to that found when ^14^C-Dp44mT and HSA were incubated together (Fig. 2B). Incubation of HSA with unlabeled Dp44mT or PIH had no significant effect on ^14^C-Bp4eT release from HSA (Fig. 2B). However, incubating HSA with an excess of unlabeled Dp44mT or Bp4eT significantly (*p*<0.001-0.05) increased the release of ^14^C-PIH from the HSA-containing dialysis sac relative to that found with HSA alone (Fig. 2B). This latter finding suggests these drugs possess common binding sites and inhibit PIH from binding to HSA, or alternatively, Dp44mT and Bp4eT bind at distant sites which may then allosterically influence ^14^C-PIH-binding. In conclusion, competition experiments revealed that HSA has some common and/or interacting sites for these ligands.

### Computational Docking Studies

Molecular docking studies were then performed to further characterize the ligand-binding sites on HSA (Supplementary Fig. 1A-B). Warfarin (Supplementary Fig. 1Ai) and ibuprofen (Supplementary Fig. 1Bi) were also docked and complexed to HSA as relevant controls, considering that their binding to Sudlow's site I and II, respectively, are well established [5]. The best docking poses of warfarin and ibuprofen correctly reproduced the experimental bioactive conformations with a root mean squared deviation of less than 1 Å from that of the ligand pose present in the X-ray structure (PDB code: 2BXD and 2BXG, respectively).

### Docking at Sudlow's Site I of HSA

These simulations docked warfarin, Dp44mT, Bp4eT and PIH at Sudlow's site I of HSA (Supplementary Fig. 1Ai-iv). Both warfarin and PIH made H-bonds with HSA (Supplementary Fig. 1Ai, iv), whereas Dp44mT and Bp4eT predominantly made hydrophobic and van der Waals interactions (Supplementary Fig. 1Aii-iii). The phenyl ring of warfarin resulted in π–π stacking with Phe211 and Trp214 and cation-π interactions with Lys199, which underlie its high binding affinity (Supplementary Fig. 1Ai). The docking of PIH (Supplementary Fig. 1Aiv) showed H-bonds with Tyr150, Arg222, Arg257 and Ala291. The hydrophobic groups of Dp44mT (Supplementary Fig. 1Aii) and Bp4eT (Supplementary Fig. 1Aiii) were favorably enclosed by the hydrophobic regions on HSA. Interestingly, the orientation of Bp4eT was flipped compared to Dp44mT when docked into Sudlow's site I, despite these two ligands having similar structures (Fig. 1A). This effect may due to the formation of a minimum energy conformation by Bp4eT under this pose [46].

As an approximation of the interaction of the ligand with the protein in terms of relative binding affinity, the GlideScore or GScore (GS; or docking score) was calculated from the docking simulations [46]. Docking of the agents at Sudlow's site I demonstrated the following GS order: warfarin (GS = −8.40 kcal/mol) > Bp4eT (GS = −8.15 kcal/mol) > PIH (GS = −8.03 kcal/mol) > Dp44mT (GS = −7.21 kcal/mol). These data suggest Dp44mT has the least affinity for Sudlow's site I. However, the GS indicates that Bp4eT and PIH have a similar affinity for Sudlow's site I. Notably, the GS parameter is an estimate only, and as the GS values are similar for Bp4eT and PIH, it should not be used to rank these ligands in terms of relative binding affinity.

### Docking at Sudlow's Site II of HSA

Ibuprofen, Dp44mT, Bp4eT and PIH were also virtually docked at Sudlow's site II of HSA (Supplementary Fig. 1Bi-iv). In the X-ray structure of HSA and ibuprofen [47], ibuprofen interacts with Arg410, Tyr411 and Lys414 *via* H-bonds. These interactions were correctly modeled with an additional cation-π interaction between Arg410 and the phenyl ring of ibuprofen (Supplementary Fig. 1Bi). PIH formed H-bonds to Arg410 (2.07 Å) and Tyr411 (2.18 Å) through its hydroxymethyl and hydroxyl groups, respectively (Supplementary Fig. 1Biv). The distal parts of the molecule were mainly located in a hydrophobic pocket, in a similar fashion to that of ibuprofen (*cf.* Supplementary Fig. 1Bi to 1Biv). Interestingly, while Dp44mT and Bp4eT both bind in the same putative binding pocket as PIH, a different ligand orientation was observed. The main interactions were H-bonds formed from the pyridyl nitrogen acceptor to Asn391 and cation-π interactions of the second pyridyl ring with Lys414 (Supplementary Fig. 1Bii-iii).

The GS obtained after docking the ligands at Sudlow's site II demonstrated the following order, namely: ibuprofen (GS = −8.50 kcal/mol) > PIH (GS = −6.0 kcal/mol) > Bp4eT (GS = −5.0 kcal/mol) > Dp44mT (GS = −4.9 kcal/mol). Again, Dp44mT showed the lowest interaction with Sudlow's site II, which was consistent with the lack of effect of this ligand in competition studies performed using equilibrium dialysis experiments (Fig. 2A). On the other hand, PIH showed the highest affinity for Sudlow's site II, relative to Bp4eT and Dp44mT, which was consistent with the dialysis studies assessing competition with ibuprofen (Fig. 2A).

In conclusion, molecular modeling indicated that ^14^C-PIH binds to HSA at both Sudlow's site I and II, potentially *via* H-bonds and this was consistent with the competition studies with warfarin and ibuprofen in dialysis experiments (Fig. 2A). Molecular modeling suggested that ^14^C-Bp4eT may share these HSA-binding sites, although in dialysis studies (Fig. 2A), limited competition was observed with warfarin only, presumably at Sudlow's site I. Dp44mT had the weakest interaction with Sudlow's site I and II, which was in agreement with its lack of effect in competition studies with warfarin and ibuprofen, respectively (Fig. 2A).

### HSA Markedly Increases ^14^C-Dp44mT Uptake, But Decreases ^14^C-Bp4eT and ^14^C-PIH Uptake

**Figure 3:**
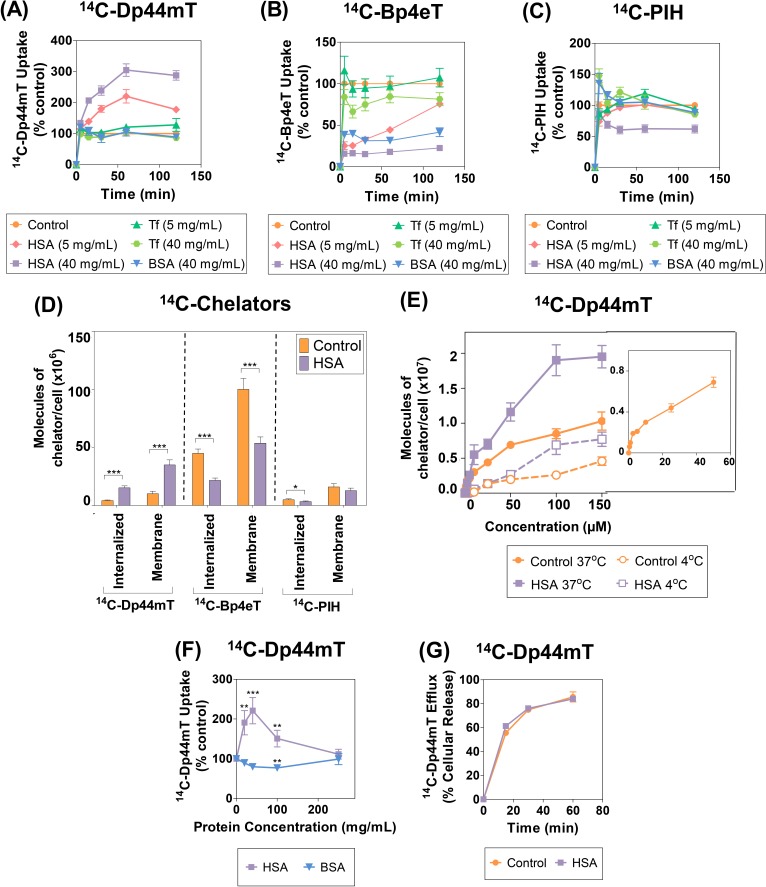
(A-C): Effect of human serum albumin (HSA), bovine serum albumin (BSA) and transferrin (Tf) on the uptake of: (A) ^14^C-Dp44mT; (B) ^14^C-Bp4eT; and (C) ^14^C-PIH by the human SK-N-MC neuroepithelioma cell line at 37°C The cells were incubated in media containing the ^14^C-chelator (25 μM) in the presence and absence of HSA (5 or 40 mg/mL), BSA (40 mg/mL) or Tf (5 or 40 mg/mL) for 0-120 min at 37°C. The cells were then placed on ice, washed 4 times using ice-cold PBS, removed from the plates and the radioactivity was quantified. (D) HSA potentiates ^14^C-Dp44mT uptake in both the membrane and internalized compartments. SK-N-MC cells were incubated with ^14^C-Dp44mT, ^14^C-Bp4eT or ^14^C-PIH (25 μM) with or without HSA (40 mg/mL) for 2 h/37°C, washed in cold PBS, treated with Pronase (1 mg/mL) for 30 min/4°C and centrifuged at 10,000 rpm/1 min. The medium was collected to represent the Pronase-sensitive membrane-bound fraction and the cells were resuspended in PBS to represent the Pronase-insensitive internalized component. Radioactivity was quantified and results were expressed as described above. *, *p*<0.05; ***, *p*<0.001 relative to the corresponding control. (E) Stimulation of HSA uptake of ^14^C-Dp44mT is saturable. Uptake of ^14^C-Dp44mT uptake as a function of concentration in the presence or absence of HSA (40 mg/mL) by SK-N-MC cells at 37°C. SK-N-MC cells were incubated with either HSA (40 mg/mL)-containing media or protein-free media with ^14^C-Dp44mT (0.1-150 μM) for 2 h at 4°C or 37°C. The cells were then placed on ice, washed 4 times using ice-cold PBS, removed from the plates and the radioactivity quantified. Inset shows the uptake of ^14^C-Dp44mT (0.1-50 μM) by SK-N-MC cells after a 2 h/37°C incubation. (F) ^14^C-Dp44mT uptake is competitively inhibited by excess HSA. ^14^C-Dp44mT uptake in the presence of increasing concentrations of HSA or BSA at 37°C. SK-N-MC cells were incubated in media containing ^14^C-Dp44mT (25 μM) in the presence or absence of HSA or BSA (20-250 mg/mL) for 2 h/37°C. The remainder of the experiment was performed as described above in Fig. 3F. **, *p*<0.01; ***, *p*<0.001 relative to the control. (G) Efflux of ^14^C-Dp44mT from SK-N-MC cells. Cells were prelabeled with ^14^C-Dp44mT (25 μM) for 2 h/37°C in the presence or absence of HSA (40 mg/mL), washed 4 times on ice and then reincubated for 0-60 min in medium containing HSA (40 mg/mL) and the release of ^14^C-Dp44mT assessed. Results are expressed as a percentage of ^14^C-Dp44mT released (mean ± S.E.M.) of at least 3 experiments.

Considering that: (1) these ligands bind to albumin (Fig. 1B, 2A-B, Supplementary Fig. 1A-B); (2) the high levels of protein accumulation in the tumor interstitium due to the enhanced permeability and retention effect [15-17]; and (3) the potential influence of protein-drug binding on drug bioavailability [7], we examined the cellular targeting and uptake of ^14^C-Dp44mT, ^14^C-Bp4eT and ^14^C-PIH in the presence and absence of the serum proteins, HSA, BSA or Tf (Fig. 3A-C).

In these studies, concentrations of apo-Tf and HSA at 5 mg/mL and 40 mg/mL, respectively, were used to approximate their physiological concentrations in human plasma [5, 48]. ^14^C-Ligand uptake was also assessed relative to Tf at 40 mg/mL, as a direct comparison to HSA at this concentration. Similarly, HSA was used at 5 mg/mL as a comparison to physiological Tf levels (52). To examine species-specific differences in terms of the ^14^C-ligand interaction with albumin, BSA (40 mg/mL) was chosen as a control protein due to its homology with HSA [5]. Studies were initially performed using SK-N-MC cells that have been well characterized in terms of the uptake and biological activity of these ligands [24, 35, 37, 38].

Interestingly, ^14^C-Dp44mT uptake was significantly (*p*<0.001-0.05) increased in the presence of HSA (5 mg/mL and 40 mg/mL) relative to the control (*i.e.*, ligand alone in control media) at all time points examined (Fig. 3A). In contrast, addition of Tf (5 or 40 mg/mL) or BSA (40 mg/mL) led to no significant (*p*>0.05) alteration in ^14^C-Dp44mT uptake relative to the control (*i.e.,* protein-free media) after a 15-120 min incubation (Fig. 3A).

In contrast to the observations with ^14^C-Dp44mT, HSA (5 or 40 mg/mL), Tf (40 mg/mL), or BSA (40 mg/mL), markedly and significantly (*p*<0.001-0.05) reduced ^14^C-Bp4eT uptake by cells relative to the control (*i.e.*, ligand alone) at all time points (Fig. 3B). On the other hand, Tf at 5 mg/mL had no significant (*p*>0.05) effect on ^14^C-Bp4eT uptake (Fig. 3B). As observed for Bp4eT, ^14^C-PIH uptake was also significantly (*p*<0.001-0.05) reduced by HSA (40 mg/mL) after a 15-120 min incubation (Fig. 3C). Moreover, ^14^C-PIH uptake was slightly (*p*<0.05) reduced by HSA (5 mg/mL), BSA (40 mg/mL) and Tf (40 mg/mL) after 120 min relative to the control. Conversely, no overall significant (*p*>0.05) difference was evident for ^14^C-PIH uptake in Tf- containing media (5 mg/mL) compared to the control (Fig. 3C).

Collectively, the cellular uptake of ^14^C-Dp44mT was markedly increased by HSA (40 mg/mL), in contrast to Tf or BSA at the same concentration. This observation indicated that HSA, rather than other plasma proteins or albumin from another species, specifically mediated an increase in ^14^C-Dp44mT uptake. Conversely, ^14^C-Bp4eT and ^14^C-PIH uptake was reduced by HSA (40 mg/mL). These findings correlate with the appreciable binding affinity of ^14^C-Bp4eT and ^14^C-PIH for HSA (Fig. 2A), resulting in a decrease in ^14^C-Bp4eT and ^14^C-PIH uptake by SK-N-MC cells. Overall, these results indicate HSA has pronounced differential effects on ^14^C-ligand uptake despite their structural similarities (Fig. 1A).

### Membrane and Internalized Uptake of ^14^C-Dp44mT by Cells are Increased by HSA, while ^14^C-Bp4eT and ^14^C-PIH Uptake are Decreased

To determine if HSA altered the cellular distribution of the ^14^C-chelators, uptake into the internalized and membrane (non-internalized) fractions were assessed after incubation with the general protease, Pronase, using standard procedures [49-52] (Fig. 3D). HSA significantly (*p*<0.001) increased internalized and membrane uptake of ^14^C-Dp44mT by cells relative to the control (Fig. 3D). In contrast, HSA significantly (*p*<0.001) decreased the internalized and membrane uptake of ^14^C-Bp4eT compared to the control (Fig. 3D). Similarly, incubation of cells with HSA significantly (*p*<0.05) decreased internalized ^14^C-PIH uptake, while membrane uptake of ^14^C-PIH was only slightly reduced (*p*>0.05). Collectively, HSA increased the internalized and membrane uptake of ^14^C-Dp44mT, while decreasing the internalized and membrane uptake of ^14^C-Bp4eT and to a slightly lesser extent ^14^C-PIH (Fig. 3D).

### HSA Markedly Increases ^14^C-Dp44mT Uptake as a Function of Temperature and Ligand Concentration

To elucidate the mechanism involved in HSA-potentiated ^14^C-Dp44mT uptake, the temperature dependence of this process over a concentration range (0.1-150 μM) was examined during a 2 h incubation at 4°C or 37°C in the presence or absence of HSA (40 mg/mL) using SK-N-MC cells (Fig. 3E). In the absence of HSA, ^14^C-Dp44mT uptake at 37°C saturated at approximately 5-10 μM (see inset Fig. 3E), as reported previously, suggesting the presence of a putative Dp44mT receptor/carrier [34]. The addition of HSA significantly (*p*<0.001-0.01) increased ^14^C-Dp44mT uptake at 37°C at ligand concentrations greater than ≥25 μM (Fig. 3E). Saturation of the HSA-stimulated uptake mechanism occurred at a Dp44mT concentration of 100 μM (Fig. 3E). These two saturation events suggest two different Dp44mT-binding sites in the presence or absence of HSA.

Examining ^14^C-Dp44mT uptake in the presence of HSA using non-linear regression analysis demonstrated a high correlation (*r^2^* = 0.97) and resulted in a *B_max_* value of 2.92 ± 0.12 × 10^7^ molecules/cell (*n* = 9) and a *K_d_* value of 65 ± 6 μM (*n* = 9). In the absence of HSA, non-linear regression also indicated a high correlation (*r^2^* = 0.96), but resulted in a lower *B_max_* value of 1.20 ± 0.04 × 10^7^ molecules/cell (*n* = 9) and a lesser *K_d_* value of 33 ± 3 μM (*n* = 9). Of relevance, in a previous study using SK-N-MC cells under different experimental conditions, a higher *B_max_* value (4.28 × 10^7^ molecules of chelator/cell) and a lower *K_d_* value (2.45 μM) were observed for Dp44mT uptake in the absence of HSA [34]. These dissimilar results are probably due to the presence of 10% (v/v) FCS in the earlier study [34], which is known to affect cellular metabolism, receptor dynamics and expression [49, 50, 53].

As demonstrated previously in the absence of HSA [34], ^14^C-Dp44mT uptake as a function of concentration was temperature-dependent, with a significant (*p*<0.001) decrease in cellular ^14^C-Dp44mT uptake being observed at 4°C relative to 37°C at all concentrations (Fig. 3E). At 37°C, the cell is metabolically active and results in receptor recycling [51, 54, 55]. Thus, the ^14^C-ligand can label receptors at both the plasma membrane and those cycling intracellularly [51, 54, 55]. In contrast, at 4°C, cells are metabolically inactive, inhibiting receptor cycling. Thus, only plasma membrane-bound receptors are labeled with the ligand, leading to a decrease in uptake relative to that found at 37°C [51, 54, 55].

Notably, ^14^C-Dp44mT uptake as a function of concentration in the presence of HSA was also temperature-dependent. In fact, a significant (*p*<0.001-0.01) decrease in ^14^C-Dp44mT uptake in the presence of HSA was observed at 4°C relative to 37°C at all Dp44mT concentrations (Fig. 3E). The saturable and temperature-dependent nature of ^14^C-Dp44mT uptake in the presence of HSA suggested a mechanism consistent with a carrier/receptor-mediated uptake process.

### Excess Non-Physiological Levels of Unlabeled HSA Decrease Dp44mT Uptake by Cells

Next, the effect of increasing HSA and BSA concentrations (20-250 mg/mL) on ^14^C-Dp44mT uptake by SK-N-MC cells was examined after 2 h/37°C (Fig. 3F). These studies were performed to determine the ability of excess HSA levels to compete with and inhibit the enhanced HSA-mediated uptake of ^14^C-Dp44mT. Furthermore, considering BSA (40 mg/mL) did not increase ^14^C-Dp44mT uptake (Fig. 3A), relevant control studies were also performed using the same concentrations of BSA (20-250 mg/mL; Fig. 3F).

As evident in Fig. 3A, BSA did not significantly (*p*>0.05) increase ^14^C-Dp44mT uptake at all concentrations tested (Fig. 3F). In fact, BSA (100 mg/mL) significantly (*p*<0.01) decreased ^14^C-Dp44mT uptake to 77 ± 4% of the control (*i.e.*, ^14^C-Dp44mT in the absence of protein; Fig. 3F). In contrast, as observed in Fig. 3A and E, ^14^C-Dp44mT uptake was significantly (*p*<0.001-0.01) increased in the presence of HSA (20-100 mg/mL) relative to the control (*i.e.*, ^14^C-Dp44mT in the absence of protein; Fig. 3F). However, after the pronounced increase in ^14^C-Dp44mT uptake up to the physiological HSA concentration in plasma (40 mg/mL), ^14^C-Dp44mT uptake then decreased as the HSA concentration increased up to 100 and 250 mg/mL (Fig. 3F). In fact, at this latter HSA concentration, a marked and significant (*p*<0.01) decrease in ^14^C-Dp44mT uptake was observed in comparison to physiological HSA levels (40 mg/mL; Fig. 3F). Thus, it can be speculated that the decrease in ^14^C-Dp44mT uptake at higher HSA concentrations relative to physiological levels may be due to the ability of excess HSA to compete with ^14^C-Dp44mT-bound HSA for the cellular HSA-binding site.

### Effect of HSA on ^14^C-Dp44mT Efflux from Cells

We also investigated the effect of HSA on efflux of ^14^C-Dp44mT (Fig. 3G), as the stimulatory effects of HSA on intracellular uptake of ^14^C-Dp44mT (Fig. 3D) could also be due to its effect on the release of the ligand from the cell. For example, decreased efflux of ^14^C-Dp44mT from cells in the presence of HSA could lead to cellular accumulation of the ligand. To assess this hypothesis, SK-N-MC cells were pre-labeled with ^14^C-Dp44mT (25 μM) in the presence or absence of HSA (40 mg/mL) for 2 h at 37°C. To replicate physiological conditions, the cellular efflux of ^14^C-Dp44mT was then assessed in the presence of HSA (40 mg/mL) as a function of time (0-60 min) at 37°C.

The cellular release of ^14^C-Dp44mT increased with time and reached a plateau at 30 min, where 74.8 ± 2.3% of ^14^C-Dp44mT was released (Fig. 3G). Importantly, the pre-labeling of cells with ^14^C-Dp44mT in the presence of HSA did not significantly (*p*>0.05) alter the efflux of ^14^C-Dp44mT at all time points (Fig. 3G). Performing the efflux incubation in the absence of HSA also demonstrated no difference in ^14^C-Dp44mT release when cells were labeled in the presence or absence of HSA (data not shown). In summary, these data indicate that other factors, besides the efflux of the ligand, were responsible for the enhanced cellular uptake of ^14^C-Dp44mT in the presence of HSA.

### The Increase in ^14^C-Dp44mT Uptake Mediated by HSA is Observed in a Variety of Neoplastic and Normal Cell-Types

To understand if the increase of ^14^C-Dp44mT uptake or inhibition of ^14^C-Bp4eT and ^14^C-PIH uptake mediated by HSA were specific to certain cell-types, ^14^C-chelator uptake in the presence and absence of HSA was examined using a range of cells (Supplementary Fig. 2A-C). The uptake of the ^14^C-chelators was examined in a variety of immortal cancer/transformed cell-types (*i.e.,* SK-N-MC neuroepithelioma, SK-Mel-28 melanoma, MCF-7 breast cancer, DMS-53 lung carcinoma, HepG2 hepatoma, HK-2 immortalized kidney proximal tubule epithelial cells) and normal, mortal cells (*i.e.,* HUVECs and MRC-5 fibroblasts) in HSA-containing (40 mg/mL) or protein-free media for 2 h/37°C (Supplementary Fig. 2A-C).

As evident in Fig. 3A, in SK-N-MC cells, ^14^C-Dp44mT uptake was significantly (*p*<0.01) increased by HSA to 300 ± 10% of the control (*i.e.*, ligand without HSA; Supplementary Fig. 2A). Additionally, HSA also significantly (*p*<0.01-0.001) increased ^14^C-Dp44mT uptake in SK-Mel-28, MCF-7, HUVEC and DMS-53 cells to 169-372% of the control, demonstrating that this effect was not specific to SK-N-MC cells (Supplementary Fig. 2A). Interestingly, HSA did not significantly (*p*>0.05) increase uptake of ^14^C-Dp44mT in MRC-5, HepG2 and HK-2 cells compared to the control (Supplementary Fig. 2A). In fact, HSA slightly, but significantly (*p*<0.01), decreased ^14^C-Dp44mT uptake by HK-2 cells *versus* the control (Supplementary Fig. 2A).

In contrast to ^14^C-Dp44mT, ^14^C-Bp4eT uptake was significantly (*p*<0.01-0.001) decreased in the presence of HSA (40 mg/mL) in all cell-types studied (Supplementary Fig. 2B). Similarly to ^14^C-Bp4eT, ^14^C-PIH uptake was also significantly (*p*<0.01-0.05) inhibited in the presence of HSA (40 mg/mL) in all cell-types examined, except for DMS-53, MRC-5 and HK-2 cells, where a non-significant (*p*>0.05) decrease was observed (Supplementary Fig. 2C). Hence, in contrast to ^14^C-Dp44mT, these results indicated that the ability of HSA to inhibit ^14^C-Bp4eT or ^14^C-PIH uptake was independent of the cell-type assessed.

Collectively, these studies demonstrated that the HSA-mediated increase in ^14^C-Dp44mT uptake and decrease in ^14^C-Bp4eT and ^14^C-PIH uptake was observed in a variety of normal and neoplastic cell-types. Considering this, albumin receptors/binding sites have been previously reported in a variety of cell-types [36]. Hence, we examined the expression of five known albumin receptors/binding proteins, namely: calreticulin [56], hnRNP [56], cubilin [57], SPARC [58] and FcRn [59] in the immortal and normal/mortal cell lines assessed above. However, no direct correlation was observed between the expression of these proteins (data not shown) and HSA-mediated ^14^C-Dp44mT uptake by these cell-types (Supplementary Fig. 2A). This observation suggested that HSA-mediated Dp44mT uptake was independent of these albumin-binding proteins.

### HSA Specifically Binds to Cells, but Dp44mT, Bp4eT or PIH, do not Affect ^125^I-Labeled HSA Uptake

**Figure 4:**
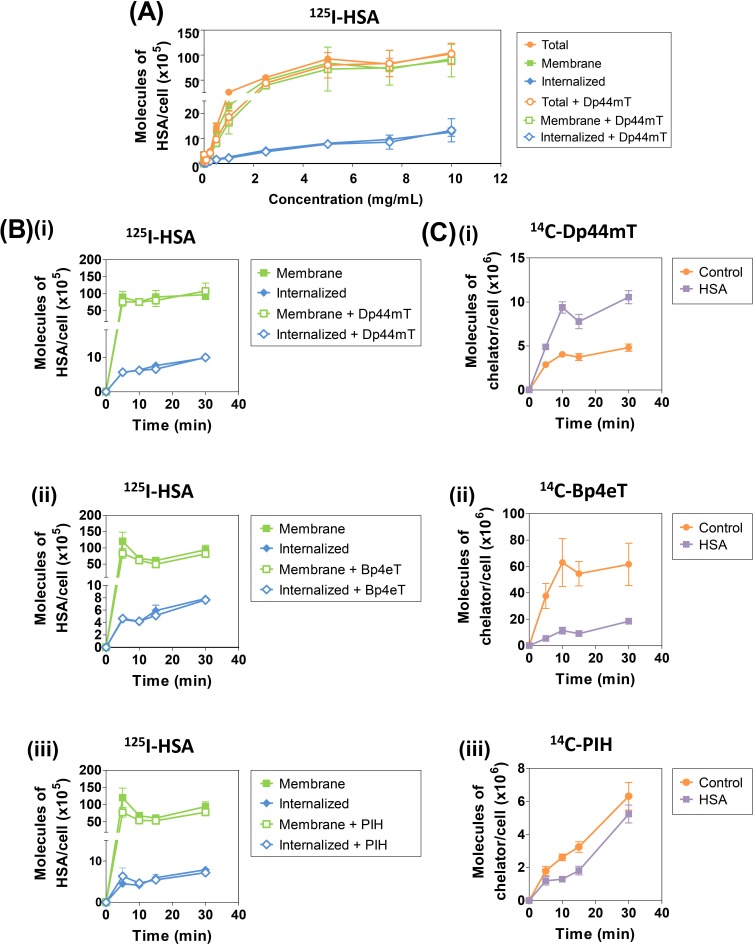
(A) Dp44mT has no effect on the uptake of ^125^I-HSA by human SK-N-MC cells as a function of concentration at 37°C SK-N-MC cells were incubated in media with ^125^I-HSA (0.001-10 mg/mL) in the presence and absence of unlabeled Dp44mT (25 μM) for 2 h/37°C. Cells were washed in cold PBS, treated with Pronase (1 mg/mL) for 30 min/4°C and radioactivity of the resulting Pronase-sensitive (membrane-bound) fraction and the Pronase-insensitive (cellular fraction) was assessed. Results are expressed as mean ± S.E.M. from 3 experiments. (B) Effect of unlabeled (i) Dp44mT, (ii) Bp4eT, or (iii) PIH on the uptake of ^125^I-HSA by SK-N-MC cells as a function of time at 37°C. SK-N-MC cells were incubated with ^125^I-HSA (7.5 mg/mL) with or without unlabeled Dp44mT, Bp4eT, or PIH (25 μM) for 30 min/37°C. Subsequent steps were performed as above. (C) Effect of HSA (7.5 mg/mL) on (i) ^14^C-Dp44mT, (ii) ^14^C-Bp4eT or (iii) ^14^C-PIH (25 μM) uptake by SK-N-MC cells as a function of time at 37°C. Experiments were performed in parallel to those in Figure 6B, with the methodology being the same as that described in Figure 4A-C.

To further elucidate the mechanisms behind the potentiation of ^14^C-Dp44mT targeting by HSA, the cellular uptake of ^125^I-labeled HSA by SK-N-MC cells was examined in the presence and absence of unlabeled Dp44mT, Bp4eT or PIH (25 μM; Fig. 4A-B). It was hypothesized that cellular stress induced by Dp44mT may increase ^125^I-HSA-mediated uptake, and thus, potentiate the transport of the chelator into the cell. In these studies, the uptake of ^125^I-HSA (0.001-10 mg/mL) was performed as a function of concentration after a 2 h/37°C incubation with SK-N-MC cells in the presence and absence of Dp44mT (Fig. 4A).

The total uptake of ^125^I-HSA by SK-N-MC cells plateaued at approximately 5-7.5 mg/mL and occurred by a single exponential process, suggesting a saturable binding site (*B_max_*: 1.46 ± 0.10 × 10^7^ molecules/cell; *K_d_*: 62 ± 11 μM; Fig. 4A). Of note, previous studies have identified *K_d_* values for albumin-binding sites in the micomolar range (0.25 - 15.1 μM) in other cell-types [60, 61]. The internalized (Pronase-insensitive) and membrane (Pronase-sensitive) uptake of ^125^I-HSA also increased as a function of ^125^I-HSA concentration and again plateaued at approximately 5-7.5 mg/mL (Fig. 4A). These observations suggested a saturable membrane-binding site which became internalized, potentially by a process of receptor-mediated endocytosis, which has been described previously for HSA receptors [36, 62, 63]. Notably, only a fraction of ^125^I-HSA (7.5 mg/mL) was internalized, with approximately 90% remaining membrane-bound (Fig. 4A). Additionally, the internalized or membrane-bound ^125^I-HSA uptake was not significantly (*p*>0.05) altered in the presence of Dp44mT (Fig. 4A).

To further elucidate the differential effects of HSA on ligand uptake, ^125^I-HSA (7.5 mg/mL) uptake was examined in the presence of unlabeled Dp44mT, Bp4eT and PIH (25 μM) as a function of time (5-30 min; Fig. 4Bi-iii). This concentration of HSA was utilized as uptake became clearly saturated at this concentration (Fig. 4A). In parallel with these studies, the uptake of ^14^C-Dp44mT, ^14^C-Bp4eT and ^14^C-PIH (25 μM) was performed as a function of time (5-30 min) in the presence or absence of HSA (7.5 mg/mL) to assess the effect of this protein on ^14^C-chelator uptake (Fig. 4Ci-iii). Irrespective of the presence or absence of the ligands, HSA uptake was biphasic, consisting of a rapid increase in internalized (Pronase-insensitive) and membrane-bound (Pronase-sensitive) ^125^I-HSA uptake followed by a plateau after 5 min of incubation (Fig. 4Bi-iii). These kinetics are consistent with the initial binding of ^125^I-HSA to its receptor, uptake by endocytosis, followed by release of the ligand by exocytosis, as described for other plasma proteins in other neoplastic cell-types [50]. As found for ^125^I-HSA uptake as function of concentration (Fig. 4A), membrane-bound ^125^I-HSA was markedly greater than the internalized ^125^I-HSA uptake, with approximately 10% of the total ^125^I-HSA being internalized (8.5 × 10^6^ molecules of HSA/cell; Fig. 4Bi-iii). The internalized or membrane-bound ^125^I-HSA uptake was not significantly (*p*>0.05) altered in the presence of Dp44mT, Bp4eT, or PIH (Fig. 4Bi-iii).

As shown previously (Fig. 3A), ^14^C-Dp44mT uptake was significantly (*p*<0.001) increased in the presence of unlabeled HSA (7.5 mg/mL) at all time points examined (Fig. 4Ci). Moreover, as evident in Fig. 3B and C, unlabeled HSA significantly (*p*<0.05) decreased ^14^C-Bp4eT and ^14^C-PIH uptake relative to the control (Fig. 4Cii-iii). Collectively, these studies demonstrated the altered uptake of the ^14^C-chelators in the presence of HSA was not due to altered HSA uptake.

### Effect of Glucose-Deprivation, Metabolic and Endocytosis Inhibitors, Temperature and Lysosomotropic Agents on Dp44mT Uptake in the Presence and Absence of HSA

To differentiate between the cellular mechanisms involved in ^14^C-Dp44mT uptake in the presence and absence of HSA, a series of conditions were utilized examining: (1) glucose-deprivation and several well characterized metabolic inhibitors [34, 35]; (2) incubation temperature; (3) an endocytosis inhibitor [64-67]; (4) and lysosomotropic agents [52, 65, 68]. The effects of these agents on ^14^C-Dp44mT uptake were compared to parallel experiments examining uptake of ^125^I-HSA or the positive control, ^59^Fe-^125^I-Tf. This comparison was performed as ^59^Fe-^125^I-Tf is well known to be internalized by receptor-mediated endocytosis in SK-N-MC cells and many other cell-types [52, 69-71]. In terms of ^59^Fe-^125^I-Tf uptake, we have assessed both ^59^Fe bound to the specific binding sites of the protein (^59^Fe-Tf), as well as the protein itself (^125^I-Tf; [52, 69-71]).

#### (1) Effect of Glucose-Deprivation and Metabolic Inhibitors on ^14^C-Dp44mT Uptake

We previously demonstrated that ^14^C-Dp44mT uptake by SK-N-MC cells was dependent on ATP synthesis *via* oxidative phosphorylation in the absence of HSA, as they could be partly inhibited using the metabolic inhibitors, sodium azide (30 mM), or sodium cyanide (5 mM) [34], which inhibit complex IV of the mitochondrial electron transport chain [72]. In this investigation, the same protocol was used as described in [34], in which cells were preincubated with inhibitors for 30 min/37°C, followed by the addition of ^14^C-Dp44mT or ^14^C-Dp44mT and HSA to these solutions, which were then incubated with the cells for 1 h/37°C. We previously demonstrated that these same incubation conditions with inhibitors markedly suppressed cellular ATP levels [34] that are vital for many cellular processes *e.g.*, endocytosis [52, 70, 73].

**Figure 5:**
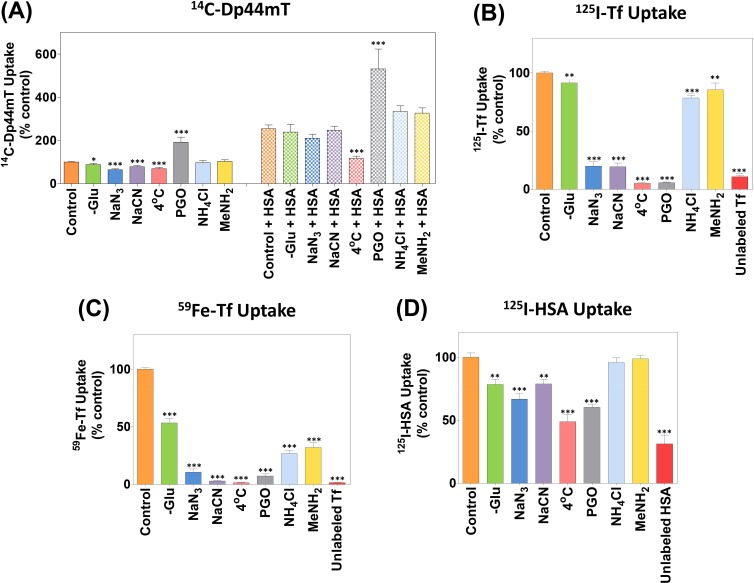
The effect of metabolic and endocytosis inhibitors, temperature, lysosomotropic agents or a 100-fold excess of protein on the uptake of ^14^C-Dp44mT in the presence and absence of HSA, or ^59^Fe-^125^I-Tf or ^125^I-HSA uptake SK-N-MC cells were pre-incubated with: (1) FCS-free media; (2) FCS- and glucose-free media (−Glu); (3) FCS- and Glu-free media containing the known metabolic inhibitors sodium azide (30 mM) or sodium cyanide (5 mM); (4) FCS-free media at 4°C; (5) FCS-free media containing the endocytosis inhibitor, phenylglyoxal (PGO; 5 mM); (6) FCS-free media containing the lysosomotropic agents, ammonium chloride (15 mM) or methylamine (15 mM); or (7) 100-fold excess unlabeled Fe-Tf or HSA (75 μM) for 30 min at 37°C unless otherwise stated. Following this, the uptake of (A) ^14^C-Dp44mT (25 μM) in the presence or absence of HSA (40 mg/mL), (B-C) ^59^Fe-^125^I-Tf (0.75 μM) or (D) ^125^I-HSA (0.75 μM) by cells was assessed under the continuation of these 7 incubation conditions for 1 h. The cells were then washed and processed for quantification. Results are expressed as mean ± S.E.M. (3 experiments). *, *p*<0.05; **, *p*<0.01; ***, *p*<0.001 relative to the corresponding control.

Incubation of cells in the absence of glucose (−Glu) led to a slight, but significant (*p*<0.05) decrease in ^14^C-Dp44mT uptake relative to cells incubated with glucose-containing medium (*i.e.*, control; Fig. 5A). Similarly to previous studies examining ^14^C-Dp44mT uptake in the absence of HSA [34], herein we also show that in glucose-free medium containing sodium azide or sodium cyanide, ^14^C-Dp44mT uptake was significantly (*p*<0.001) reduced to 65 ± 4% and 78 ± 6%, respectively, relative to glucose-containing control medium (Fig. 5A). In contrast, no significant (*p*>0.05) difference in ^14^C-Dp44mT uptake in the presence of HSA *versus* the control was shown using glucose-free medium (−Glu) or these inhibitors (Fig. 5A). These data suggest HSA-mediated ^14^C-Dp44mT uptake was less reliant on metabolic energy relative to ^14^C-Dp44mT uptake alone.

#### (2) Effect Incubation Temperature on ^14^C-Dp44mT Uptake

Additional studies demonstrated that ^14^C-Dp44mT uptake in the absence of HSA was significantly (*p*<0.001) reduced at 4°C relative to 37°C (Fig. 5A). A similar and significant (*p*<0.001) inhibitory effect of incubation at 4°C was also observed on HSA-mediated ^14^C-Dp44mT uptake relative to uptake observed at 37°C (Fig. 5A). Hence, ^14^C-Dp44mT uptake in the presence and absence of HSA was dependent on incubation temperature.

#### (3 & 4) Effect of an Endocytosis Inhibitor and Lysosomotropic Agents on ^14^C-Dp44mT Uptake

Experiments then assessed the role of endocytosis and endosomal/lysosomal acidification on ^14^C-Dp44mT uptake in the presence and absence of HSA. In these studies, the well-characterized endo-/exocytosis inhibitor, phenylglyoxal (PGO; 5 mM; [64-66]), or the well known lysosomotropic agents, ammonium chloride (15 mM) or methylamine (15 mM), were assessed [52, 65, 68]. After incubation with PGO, there was a significant (*p*<0.001) increase in ^14^C-Dp44mT uptake relative to the control in the presence or absence of HSA (Fig. 5A). Considering that PGO inhibits both endocytosis and exocytosis [64-66], it can be speculated that the PGO-enhanced accumulation of ^14^C-Dp44mT was due to the inhibition of exocytosis, thereby preventing efflux of this ligand.

In contrast, both lysosomotropic agents had no significant (*p*>0.05) effect on ^14^C-Dp44mT uptake in the presence or absence of HSA relative to the control (Fig. 5A). Considering the failure of PGO and lysosomotropic agents to inhibit ^14^C-Dp44mT uptake with or without HSA, and the fact that they significantly (*p*<0.001-0.01) inhibit ^59^Fe- and ^125^I-Tf uptake from ^59^Fe-^125^I-Tf (see Fig. 5B, C), these data suggest that ^14^C-Dp44mT uptake was independent of endocytosis and the acidification of the endosomal/lysosomal compartment.

### Effect of Glucose-Deprivation, Metabolic and Endocytosis Inhibitors, Temperature and Lysosomotropic Agents on ^59^Fe-^125^I-Transferrin and ^125^I-HSA Uptake

In parallel studies implementing the same incubation conditions as the ^14^C-Dp44mT uptake experiments (Fig. 5A), the cellular uptake of ^59^Fe-^125^I-Tf (0.75 μM; Fig. 5B-C) or ^125^I-HSA (0.75 μM; Fig. 5D) were investigated, as uptake of these proteins are well characterized [52, 65, 70, 73]. The uptake of ^59^Fe-^125^I-Tf and ^125^I-HSA by cells was significantly (*p*<0.001-0.01) inhibited in the absence of glucose relative to media containing glucose (control; Fig. 5B-D). This effect of glucose-free medium was generally potentiated in the presence of sodium azide and sodium cyanide, decreasing ^59^Fe-Tf, ^125^I-Tf and ^125^I-HSA uptake to 4-14%, 23-24% and 67-79% of the control, respectively (Fig. 5B-D). Hence, similarly to ^14^C-Dp44mT uptake (Fig. 5A), ^59^Fe-^125^I-Tf and ^125^I-HSA uptake was dependent on mitochondrial electron transport chain activity (Fig. 5B-D). In contrast, this was markedly different to ^14^C-Dp44mT uptake in the presence of HSA (Fig. 5A), that was independent of the inhibition of mitochondrial electron transport chain.

Cells incubated at 4°C internalized significantly (*p*<0.001) less ^59^Fe-Tf, ^125^I-Tf, or ^125^I-HSA than those incubated at 37°C (Fig. 5B-D). Incubation of cells with the endo-/exocytosis inhibitor, PGO, or the lysosomotropic agents, ammonium chloride or methylamine, markedly and significantly (*p*<0.001-0.01) inhibited ^125^I-Tf (Fig. 5B) and ^59^Fe-Tf uptake (Fig. 5C). Similarly, the uptake of ^125^I-HSA was significantly (*p*<0.001) inhibited in the presence of PGO (Fig. 5D). This observation suggests that ^14^C-Dp44mT enters cells independently of ^125^I-HSA, as ^14^C-Dp44mT uptake in the presence or absence of HSA was significantly (*p*<0.001) increased upon incubation with PGO (Fig. 1A). In contrast to ^59^Fe-Tf or ^125^I-Tf uptake, ^125^I-HSA uptake was not significantly (*p*>0.05) altered in the presence of the lysosomotropic agents, ammonium chloride or methylamine (Fig. 5D). Importantly, ^59^Fe-Tf, ^125^I-Tf, or ^125^I-HSA uptake was significantly (*p*<0.001) inhibited upon the addition of a 100-fold excess of the unlabeled protein, namely Fe-Tf or HSA, respectively (Fig. 5B-D), suggesting competition between the unlabeled and labeled protein for the same binding sites. These results agree with previous studies demonstrating ^59^Fe-Tf, ^125^I-Tf and ^125^I-HSA uptake occur *via* energy- and temperature-sensitive endocytosis [69, 70, 74, 75].

Together, these data indicate in contrast to ^59^Fe-Tf, ^125^I-Tf and ^125^I-HSA uptake by cells, ^14^C-Dp44mT uptake in the presence of HSA was insensitive to glucose levels, inhibition of energy metabolism and the suppressive effects of lysosomotropic agents or an endo-/exocytosis inhibitor. These observations suggested the HSA-stimulated mechanism of ^14^C-Dp44mT uptake occurred by a different pathway to the uptake of either ^125^I-HSA or ^59^Fe-^125^I-Tf that occur by endocytosis or endocytosis requiring endosomal acidification, respectively [52, 69-71, 74, 75].

### HSA Enhances the Anti-Proliferative Activity of Dp44mT and Inhibits that of Bp4eT and PIH

**Figure 6:**
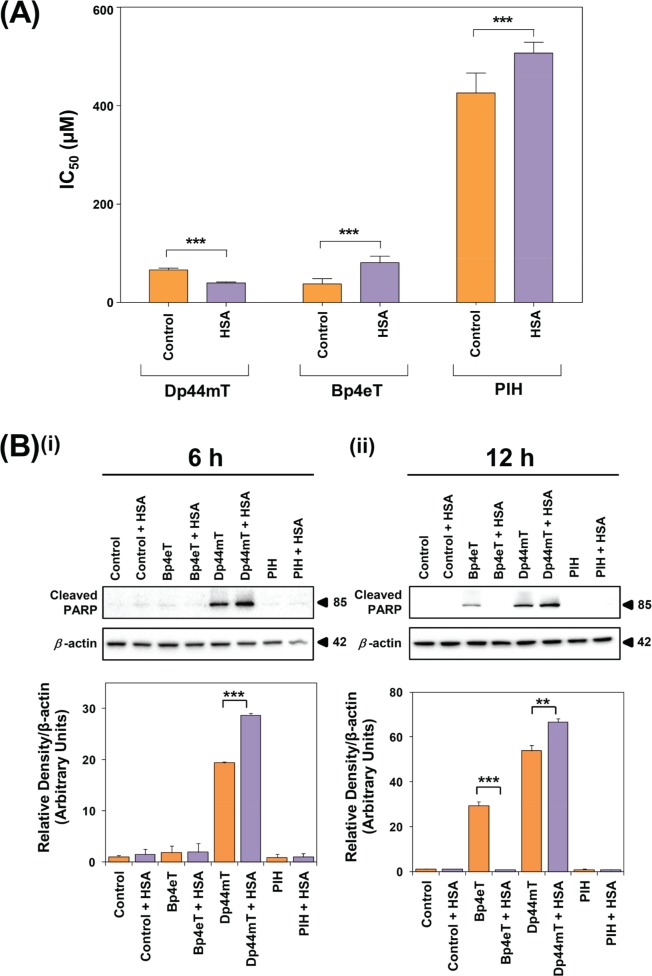
The effect of HSA on the anti-proliferative and apoptotic activity of Dp44mT, Bp4eT and PIH (A) The anti-proliferative activity of Dp44mT, Bp4eT and PIH in the presence of HSA for 24 h/37°C. Cells were incubated with Dp44mT (30-120 μM), Bp4eT (30-120 μM), PIH (150-600 μM) or vehicle alone (control) in the presence or absence of HSA (40 mg/mL) for 24 h/37°C. Trypan blue was used to obtain direct cell counts and to determine IC_50_ values. Results are expressed as mean ± S.E.M. (3 experiments). ***, *p*<0.001 relative to the corresponding control. (B) Levels of cleaved PARP following treatment of SK-N-MC cells with Bp4eT, Dp44mT or PIH (50 μM) in the presence and absence of HSA (40 mg/mL) after (i) 6 h or (ii) 12 h/37°C. Western blots are typical of 3 independent experiments. Results are expressed as mean ± S.E.M. (3 experiments). **, *p*<0.01; ***, *p*<0.001.

Considering the increased uptake of ^14^C-Dp44mT in the presence of HSA (Fig. 3A, D, E), the effect of HSA on the anti-proliferative activity of Dp44mT was examined in SK-N-MC cells (Fig. 6A). Additionally, the effect of HSA on the anti-proliferative activity of Bp4eT and PIH were also assessed (Fig. 6A), considering the inhibitory effects of HSA on ^14^C-Bp4eT and ^14^C-PIH uptake (Fig. 3B-C). In these experiments, cells were incubated with Dp44mT (30-120 μM), Bp4eT (30-120 μM), PIH (150-600 μM) or the vehicle alone (control) in the presence or absence of HSA (40 mg/mL) for 24 h/37°C (Fig. 6A).

HSA significantly (*p*<0.001) increased the anti-proliferative activity of Dp44mT relative to Dp44mT alone, leading to a decrease in its IC_50_ (Fig. 6A). In fact, after a 24 h incubation, HSA decreased the IC_50_ of Dp44mT by ≈ 1.6-fold to 40 ± 2 μM in comparison to Dp44mT alone (66 ± 4 μM; Fig. 6A). As an additional control, the anti-proliferative activity of Dp44mT was also examined in the presence of BSA (40 mg/mL; data not shown). However, the IC_50_ of Dp44mT was not significantly (*p*>0.05) altered in the presence of BSA (IC_50_: 72 ± 2 μM) relative to the ligand alone. This observation is in agreement with our studies showing that BSA did not significantly (*p*>0.05) alter cellular ^14^C-Dp44mT uptake (Fig. 3A).

In contrast to Dp44mT, the anti-proliferative activity of Bp4eT was significantly (*p*<0.001) reduced by HSA, leading to an increase in the IC_50_ (81 ± 4 μM) relative to its activity in the absence of HSA (IC_50_: 38 ± 3 μM; Fig. 6A). Similarly, HSA significantly (*p*<0.001) increased the IC_50_ of PIH to 507 ± 7 μM compared to the ligand alone (IC_50_: 426 ± 14 μM; Fig. 6A). These data are in good agreement with our ^14^C-chelator uptake experiments and indicate that the HSA-mediated increase in ^14^C-Dp44mT uptake (Fig. 3A) results in its enhanced anti-proliferative efficacy (Fig. 6A). In contrast, the ability of HSA to decrease cellular ^14^C-Bp4eT and ^14^C-PIH uptake (Fig. 3B-C) decreased anti-proliferative activity of both ligands (Fig. 6A).

### HSA Enhances the Apoptotic Activity of Dp44mT and Inhibits that of Bp4eT

In contrast to PIH and Bp4eT, the cellular uptake and cytotoxicity of Dp44mT was enhanced in the presence of HSA (Figs. 3A, B, 6A). Thus, it was important to examine the effects of HSA on the ability of these ligands to induce apoptosis. In order to do this, the effects of Dp44mT, Bp4eT and PIH (50 μM) on the levels of the apoptotic marker, cleaved poly ADP-ribose polymerase (PARP; [76, 77]), were examined in SK-N-MC cells after a 6 or 12 h incubation at 37°C in the presence or absence of HSA (40 mg/mL; Fig. 6B).

After a 6 h incubation, Dp44mT resulted in a 19.4-fold increase in cleaved PARP levels relative to the control (Fig. 6Bi). This observation was in good agreement with the known ability of Dp44mT to induce apoptosis *in vitro* and *in vivo* [25, 78, 79]. The level of cleaved PARP upon co-incubation of Dp44mT and HSA for 6 h was significantly (*p<*0.001) increased relative to both the control and Dp44mT treatment alone (Fig. 6Bi). In fact, cleaved PARP levels were 1.3-fold greater in Dp44mT + HSA treated cells relative to Dp44mT alone. These results suggest HSA enhanced the ability of Dp44mT to induce apoptosis at this early time point. In contrast, a 6 h incubation with Bp4eT or PIH in the presence or absence of HSA did not result in significantly (*p*>0.05) increased cleaved PARP relative to their controls (Fig. 6Bi).

At the 12 h time point, Dp44mT alone and Dp44mT + HSA significantly (*p*<0.001) increased cleaved PARP relative to their controls (*i.e.*, control medium and control medium + HSA, respectively; Fig. 6Bii). Cleaved PARP levels in cells co-treated with Dp44mT + HSA showed a significant (*p*<0.01) 1.2-fold increase relative to cells treated with Dp44mT alone. Cells treated with Bp4eT alone demonstrated significantly (*p*<0.001) increased cleaved PARP relative to the control (Fig. 6Bii). However, this effect was abolished upon incubating cells with Bp4eT in the presence of HSA, resulting in cleaved PARP levels that were significantly (*p*<0.001) decreased relative to Bp4eT alone and was comparable to the control (Fig. 6Bii). Thus, HSA inhibited the ability of Bp4eT to induce cleaved PARP. In contrast, cells incubated for 12 h with PIH in the presence or absence of HSA did not result in cleaved PARP and were comparable to their controls (Fig. 6Bii).

Collectively, these results demonstrated that HSA was able to significantly (*p*<0.001-0.01) enhance the apoptotic effects of Dp44mT at 6 and 12 h and this reflected its increased cellular uptake (Fig. 3A, D, E) and anti-proliferative activity (Fig. 6A) of Dp44mT upon HSA co-treatment. In contrast, the presence of HSA was able to inhibit the apoptotic activity of Bp4eT after 12 h, which probably results from the decreased ^14^C-Bp4eT uptake observed upon incubation with HSA (Fig. 3B, D). On the other hand, PIH did not induce marked levels of PARP cleavage and this reflects the poor anti-proliferative activity of this agent [37] relative to Dp44mT [24, 25] and Bp4eT [38] (Fig. 6A).

## DISCUSSION

### Dp44mT, Bp4eT and PIH Bind to HSA

**Figure 7:**
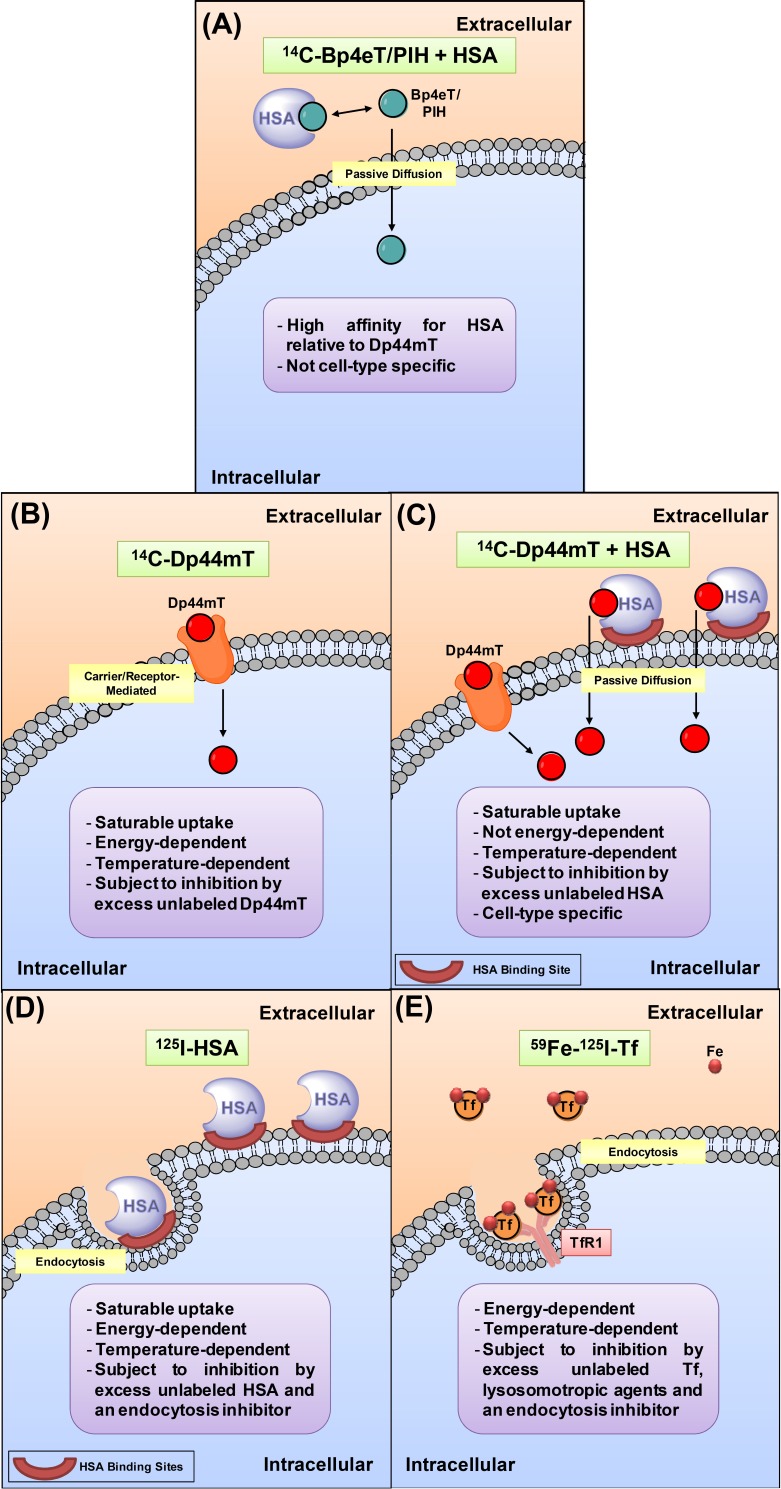
Schematic showing the internalization of ^14^C-Bp4eT/PIH or ^14^C-Dp44mT with or without HSA, ^125^I-HSA or ^59^Fe-^125^I-Tf into the cell (A) ^14^C-Bp4eT and ^14^C-PIH are transported by diffusion in the absence of HSA [34]. In the presence of HSA, HSA-binding inhibits the uptake of ^14^C-Bp4eT and ^14^C-PIH, irrespective of the cell-type. This is due to the high affinity of Bp4eT or PIH for HSA, relative to Dp44mT, reducing the levels of free drug available to diffuse into cells. (B) A different mechanism is demonstrated by the structurally similar ligand, Dp44mT. ^14^C-Dp44mT is taken up by cells *via* a receptor/carrier in the absence of HSA. This uptake process is saturable, energy- and temperature-dependent and subject to inhibition by excess unlabeled Dp44mT [34]. (C) In the presence of HSA, ^14^C-Dp44mT uptake occurs through a second, high capacity, saturable process that is cell-type specific and inhibited in the presence of excess unlabeled HSA. This process may be facilitated by: (i) a specific HSA-binding site; (ii) the fact most cellular HSA is bound to the cell membrane (rather than internalized); and (iii) the relatively low affinity of Dp44mT for HSA. These three properties facilitate Dp44mT delivery to cells and create a concentration gradient at the cell surface to enable enhanced uptake *via* dissociation and passive diffusion. The enhanced delivery of ^14^C-Dp44mT by HSA increases apoptosis and cytotoxicity. (D) ^125^I-HSA uptake by cells was saturable, temperature-dependent, inhibited by excess unlabeled HSA, and sensitive to glucose starvation and inhibitors of energy metabolism or endocytosis. Hence, this process was consistent with HSA endocytosis that is known to occur [74, 92]. (E) ^59^Fe-^125^I-Tf uptake occurs following the binding to its receptor, Tf receptor 1 (TfR1). This process was temperature- and energy-dependent, and subject to inhibition by excess unlabeled Tf, an endocytosis inhibitor, and in addition, lysosomotropic agents. Hence, ^59^Fe-^125^I-Tf uptake occurs through the well characterized process of receptor-mediated endocytosis that requires endosomal acidification [52, 68, 71, 93].

In this investigation, studies were performed to determine whether Dp44mT, Bp4eT or PIH were able to directly bind to albumin using fluorescence spectroscopy and equilibrium dialysis studies. These experiments demonstrated that all the ligands bind to HSA, although with different avidities (Fig. 1-2). In fact, equilibrium dialysis experiments indicated that ^14^C-Bp4eT became bound to HSA with similar avidity to ^14^C-PIH, while ^14^C-Dp44mT was most weakly bound to the protein (Fig. 2A). Molecular docking studies also supported these conclusions (Supplementary Fig. 1). Importantly, these findings indicating the avid binding of Bp4eT and PIH to HSA could explain the decreased uptake of these agents by cells in the presence of this protein (Fig. 3B-D). In fact, in the absence of HSA, Bp4eT and PIH are known to enter cells *via* passive diffusion [35]. Considering this, HSA may act as an extracellular ‘sink’, preventing the passive diffusion of Bp4eT and PIH into cells (Fig. 7A). Consequently, HSA did not enhance ^14^C-Bp4eT or ^14^C-PIH uptake or anti-proliferative activity, but conversely, decreased it (Fig. 3B-D, 7A).

### ^14^C-Dp44mT Uptake is Specifically Enhanced by HSA

In contrast to Bp4eT and PIH (Fig. 3B-C), Dp44mT uptake is markedly increased in the presence of physiological concentrations of HSA in the plasma (40 mg/mL) as a function of time (Fig. 3A) and concentration (Fig. 3E). In comparison, HSA markedly decreased ^14^C-Bp4eT and ^14^C-PIH uptake (Fig. 3B-C). The enhanced ^14^C-Dp44mT uptake was specific for HSA and this observation was supported by two findings. First, this effect did not occur in the presence of the same concentration of Tf, suggesting a specific interaction between Dp44mT and HSA and not other plasma proteins (Fig. 3A). Second, stimulation of Dp44mT uptake did not occur with albumin from another species, namely BSA (75.6% sequence identity to HSA; [5]), demonstrating specificity (Fig. 3A).

Previous studies from our laboratory determined that the cellular uptake of ^14^C-Dp44mT occurred *via* a saturable carrier/receptor-mediated mechanism [34] (Fig. 7B). Evidence for this mechanism was also obtained in the current investigation in the absence of HSA, with ^14^C-Dp44mT uptake saturating at 5-10 μM (Fig. 3E inset). However, the addition of HSA led to a high-capacity, saturable, uptake process with saturation occurring at a Dp44mT concentration of 100 μM (Fig. 3E). Hence, in the presence of HSA, there was evidence of an important second saturable mechanism of Dp44mT uptake. Moreover, HSA-stimulated Dp44mT uptake was inhibited by an excess of unlabeled HSA (Fig. 3F), suggesting excess HSA competes with the Dp44mT-HSA complex for HSA membrane-binding sites (Fig. 7C).

### ^14^C-Dp44mT Uptake is Increased by HSA in a Variety of Cell-Types

^14^C-Dp44mT uptake was also augmented by HSA in a variety of cancer cell lines and a non-neoplastic, mortal cell-type (Supplementary Fig. 2A). These observations demonstrated that the HSA-mediated increase of ^14^C-Dp44mT uptake was a commonly observed mechanism that was not unique to one cell-type. However, there was some cell-type specificity, and the process was not present in some neoplastic and non-neoplastic cells. This finding suggested the differential expression of HSA receptors/binding sites between cell-types. The HSA-mediated Dp44mT uptake in a variety of normal and neoplastic cells did not correlate with the expression of a panel of well known HSA receptors [36], suggesting their lack of involvement in the augmented ^14^C-Dp44mT uptake mediated by HSA.

### Mechanism of HSA-Mediated Dp44mT Uptake

Interestingly, ^125^I-HSA uptake studies indicated the presence of saturable HSA-binding sites on SK-N-MC cells (Fig. 4A), although Dp44mT, Bp4eT or PIH did not significantly affect ^125^I-HSA uptake (Fig. 4Bi-iii). Indeed, it was demonstrated that in the presence of HSA, ^14^C-Dp44mT uptake occurs through a second, high capacity, low affinity, saturable process (Fig. 3E) that is cell-type specific (Supplementary Fig. 2A) and was inhibited in the presence of excess unlabeled HSA (Fig. 3F). This uptake process had the following three features: (i) it was facilitated by a specific HSA-binding site (Fig. 3E, F); (ii) most cellular HSA was bound to the cell membrane, rather than internalized (Fig. 4A, Bi-iii); and (iii) the avidity of Dp44mT for HSA was relatively low (Fig. 2). Together, these three properties suggest Dp44mT is delivered to cells in a HSA-dependent manner that creates a concentration gradient at the cell surface that enhances subsequent uptake *via* dissociation and passive diffusion (Fig. 7C). Indeed, HSA-mediated ^14^C-Dp44mT uptake was not inhibited by glucose-deprivation, metabolic inhibitors, an endocytosis inhibitor, or lysosomotropic agents (Fig. 5A), indicating a passive uptake process (Fig. 7C), rather than active endocytosis which occurred for ^125^I-HSA (Fig. 5D, 7D) and ^59^Fe-^125^I-Tf (Fig. 5B, C, 7E).

In clear contrast to HSA-mediated ^14^C-Dp44mT uptake (Fig. 5A), ^125^I-HSA uptake by cells was reduced by glucose starvation and inhibitors of energy metabolism or endocytosis (Figs. 5D, 7D). Together, these data suggest augmentation of ^14^C-Dp44mT uptake in the presence of HSA was independent of HSA internalization. Moreover, in contrast to HSA-dependent and independent ^14^C-Dp44mT uptake (Fig. 5A), and ^125^I-HSA uptake (Fig. 5D), the uptake of ^59^Fe-^125^I-Tf occurred by the well characterized endocytic mechanism that required acidification [52, 69-71]. This was demonstrated by the inhibition of ^59^Fe-Tf uptake (Fig. 5C), and to a lesser extent ^125^I-Tf uptake (Fig. 5B), by lysosomotropic agents (Fig. 7E).

In terms of the mechanism of intracellular uptake of other molecules bound to HSA (*e.g.*, fatty acids), it has been reported that after HSA-binding to the cell membrane, fatty acid-bound albumin undergoes a conformational change [5, 11]. This alteration then results in fatty acid release in the proximity of the membrane for cellular uptake [5, 11]. The subsequent reduced affinity of albumin for the cell surface then leads to its release from the membrane [5, 11]. Similar mechanisms of transport have also been proposed for albumin-bound testosterone and tryptophan [5, 12-14]. In an analogous way to Dp44mT, the low affinity of albumin for testosterone results in the transport of this hormone from the plasma for rapid release and delivery to tissues [5, 80]. Hence, the mechanism reported in this study for HSA-mediated Dp44mT uptake, shows similar characteristics to those described for fatty acids and testosterone.

Binding of Dp44mT to HSA had the lowest relative affinity relative to Bp4eT and PIH (Fig. 2A). Hence, the relatively low affinity of Dp44mT for HSA may facilitate the release of Dp44mT for uptake by passive diffusion (Fig. 7C). In contrast, the relatively higher affinity of Bp4eT and PIH may prevent this (Fig. 7A), and thus, this may explain the differential effects of HSA observed on ligand uptake demonstrated herein. Considering this, it is notable that the ability of HSA to inhibit Bp4eT or PIH uptake was irrespective of the cell-type assessed (Supplementary Fig. 2B-C), suggesting the inhibition was independent of the cell-type. In marked contrast, the stimulation of Dp44mT uptake by HSA was dependent on cell-type (Supplementary Fig. 2A), implicating the crucial role of the cell *via* the expression of HSA-binding sites in terms of the effect observed.

### HSA Potentiates Dp44mT Targeting to Tumor Cells Resulting in Increased Anti-Proliferative and Apoptotic Activity

Significantly, HSA increased the anti-proliferative and apoptotic effects of Dp44mT (Fig. 6). Hence, the HSA-mediated increase in ^14^C-Dp44mT uptake and targeting (Fig. 3A, D, E) enhanced the anti-tumor efficacy of this drug. Conversely, the addition of HSA decreased the anti-proliferative activity of Bp4eT and PIH (Fig. 6A) and inhibited the ability of Bp4eT to induce apoptosis (Fig. 6B). This can be attributed to the HSA-induced inhibition of ^14^C-Bp4eT and ^14^C-PIH uptake (Fig. 3B-D), resulting in reduced anti-cancer efficacy of these ligands. These observations could be important for designing new therapeutics based on Dp44mT that enhance its biological efficacy. For instance, albumin-containing nanoparticles have been utilized for improving the activity of standard chemotherapeutics [17, 81, 82]. These agents utilize the enhanced permeability and retention effect and cellular uptake pathways of albumin to enhance drug permeation into tumors [17, 81-83].

Albumin nanoparticles containing the established chemotherapeutic, paclitaxel (marketed under the name Abraxane^®^), have been approved for the treatment of breast cancer, pancreatic adenocarcinoma and non-small cell lung cancer [84-86]. In fact, Abraxane^®^ is less toxic and more effective than conventional paclitaxel [84-86]. Similarly, the development of albumin nanoparticles containing thiosemicarbazones, such as Dp44mT, may enhance the delivery, anti-tumor targeting, selectivity and toxicological profile of this agent. Other thiosemicarbazone-loaded nanoparticles (known as “nanochelators”) have been examined [87], although albumin was not utilized in their composition to enhance uptake. Hence, the development of novel albumin-containing nanoparticles represents an exciting therapeutic avenue.

In conclusion, physiological levels of HSA mediate the enhanced cellular uptake and targeting of Dp44mT, resulting in increased anti-proliferative and apoptotic activity. The uptake of Dp44mT in the presence of HSA could provide therapeutic benefits by delivering greater levels of drug to cancer cells, improving its anti-tumor efficacy and tolerability.

## MATERIALS AND METHODS

### Chemicals

HSA (≥99% purity; Cat. #A8763), BSA (≥98% purity; Cat. #A7906), transferrin (Tf; ≥98% purity; Cat. #T4382), warfarin (Cat. #A2250) and ibuprofen (Cat. #I4883) were purchased from Sigma-Aldrich (St. Louis, MO). The non-radiolabeled ligands, Dp44mT, Bp4eT and PIH, were synthesized and characterized by established methods [23, 37, 38]. The ^14^C-labeled chelators, ^14^C-Bp4eT, ^14^C-Dp44mT and ^14^C-PIH, were synthesized by the Institute of Isotopes Ltd (Budapest, Hungary) and were purified and prepared as previously described [34, 35].

### Fluorescence Quenching Studies

The fluorescence spectra of HSA (2 μM) was measured with increasing concentrations of the chelators, Dp44mT, Bp4eT or PIH (0-3.67 μM), after a 2 h incubation at 37°C on a LS-55 spectrofluorometer (Perkin Elmer Life and Analytical Sciences, Waltham, MA) with a 1 cm path-length quartz cell using 15 nm/6 nm slit widths and a thermostat bath. The excitation and emission wavelengths for HSA were 295 nm and 310-450 nm, respectively, with scanning at 5 nm increments.

### Circular Dichroism

Far-UV CD data were collected using a Jasco 815 spectropolarimeter equipped with a Peltier- thermostated 6-chamber sample holder at 20°C (JASCO, Tokyo, Japan) using a 1 mm path-length quartz cell. Stock solutions of the chelators (1 mM) were prepared in ethanol. Samples containing HSA (2 μM) in the presence and absence of the chelators, Dp44mT, Bp4eT or PIH (10 μM), or the chaotrope, GndCl (Sigma-Aldrich; 6 M), were prepared in PBS and incubated at 37°C for 2 h prior to measurement. Spectra were collected at 20 nm/min over the range of 200–250 nm, with a sensitivity of 100, step size of 1 nm, digital integration time of 1 s and are the average of five scans with buffer baseline correction and background subtraction. The percentage of α-helices and β-sheets was calculated using DichroCalc [88].

### Equilibrium Dialysis Studies

Equilibrium dialysis experiments were performed using standard methods [44]. In these studies, HSA (40 mg/mL) was incubated with an excess of warfarin or ibuprofen (5 mM) or an excess of unlabeled Dp44mT, Bp4eT or PIH (0.5 mM) in PBS for 2 h/37°C in PBS to duplicate the experimental conditions utilized for uptake experiments with the ^14^C-labeled ligands (see below). The ^14^C-chelators (25 μM) were then added and the samples were further incubated for 2 h/37°C. The solutions were then placed in a dialysis membrane sac with a 12 kDa molecular weight cut-off (Sigma-Aldrich). These samples underwent dialysis in PBS and were allowed to equilibrate for 24 h/4°C on a rotating mixer. Experiments using the ^14^C-labeled ligands alone demonstrated that after this incubation period, an equilibrium was established with equal amounts of the label inside and outside of the dialysis sac. Then 1 mL aliquots were taken from the dialysate and the dialysis sac and were processed as previously described [34] using an oxidizer (Sample Oxidizer Model 307; Perkin Elmer Life and Analytical Sciences) to prevent quenching. The radioactivity in samples was measured using a Wallac 1450 MicroBeta TriLux β Counter (PerkinElmer) with appropriate calibration standards and background controls. The results were expressed as % of chelator released from the dialysis sack into the dialysate.

### Computational Docking Studies

The 2-D structures of the HSA-binding ligands were built using the Schrödinger suite (Schrödinger Inc., New York, NY, USA). Geometry minimizations were performed on all ligand conformations, with all possible ionization states at pH 7.0 ± 2.0, using the OPLS_2005 force field in MacroModel v9.8 and the Truncated Newton Conjugate Gradient. Optimizations were converged to a gradient RMSD below 0.05 kJ/mol, or continued to a maximum of 5000 iterations, at which there were negligible changes in RMSD gradients. Glide v5.8 and the extra precision scoring function were used to estimate the affinities of protein–ligand binding [89].

Initially, warfarin, Dp44mT, Bp4eT and PIH, were docked into Sudlow's site I (warfarin site), where the docking grid was defined and generated based upon the ligand-binding domain of the crystal structure of HSA in complex with warfarin (Protein Data Bank (PDB) code: 2BXD; www.rcsb.org). Similarly, the binding site for Sudlow's site II (ibuprofen site) was obtained from the co-crystal structure of HSA with ibuprofen (PDB code: 2BXG), where ibuprofen defined the centroid of the docking grid. No constraints were fixed in the active site, allowing the ligands to bind in all possible orientations. Protein preparation and refinement protocols were performed on the structure (Protein Preparation Wizard, Schrödinger).

### Cell Culture

The following human cell-types were obtained from the American Type Culture Collection (ATCC; Manassas, VA), namely DMS-53 lung carcinoma cells, SK-N-MC neuroepithelioma cells, SK-Mel-28 melanoma cells, MCF-7 breast cancer cells, MRC-5 fibroblast cells, HepG2 hepatocellular carcinoma and the papillomavirus 16 transformed kidney proximal tubule HK-2 cells. All of these cell-types, except DMS-53 lung carcinoma cells, were cultured as previously described [35] in minimum essential media (MEM; Life Technologies) at 37°C. The DMS-53 lung carcinoma cell line was cultured in a similar manner as above using Roswell Park Memorial Institute 1640 (RPMI 1640; Life Technologies). These media were supplemented with 10% (v/v) fetal calf serum (FCS; Sigma-Aldrich) and the following additives from Life Technologies: 1% (v/v) sodium pyruvate, 1% (v/v) 100× non-essential amino acids, 100 U/mL penicillin, 100 μg/mL streptomycin, 2 mM glutamine and 0.28 μg/mL fungizone. Human umbilical vein endothelial cells (HUVECs) were kindly donated by Mr. P. Pisansarakit (Heart Research Institute, Sydney, Australia) and were cultured according to established techniques [35]. All cells were cultured in an atmosphere of 5% CO_2_/95% air.

### Cellular Uptake of ^14^C-Ligands

The cellular uptake of ^14^C-chelators was performed in accordance with previously established procedures [34, 35]. Briefly, cells in culture dishes were incubated with 25 μM of ^14^C-chelator in supplement- and FCS-free media in the presence and absence of HSA (5 or 40 mg/mL), BSA (40 mg/mL) or Tf (5 or 40 mg/mL) for 0-120 min at 37°C. The cellular uptake of ^14^C-Dp44mT (0.1-150 μM) was also examined as a function of concentration in the absence or presence of HSA (40 mg/mL) at 4 or 37°C over a 2 h incubation. In studies examining the effect of proteins on ^14^C-chelator uptake as a function of protein concentration, cells were incubated in medium containing HSA or BSA (0-250 mg/mL) for 120 min/37°C. Upon completion of uptake experiments, cells were placed on ice and washed four times with ice-cold PBS. The cells were then resuspended the cells in PBS (1 mL) and Ultima Gold™ scintillation fluid was added (2.5 mL; PerkinElmer). Radioactivity was measured using the β-counter, as described above.

### Assay Examining Internalized and Membrane-Bound ^14^C-Ligand Uptake by Cells

In studies examining the internalized and membrane-bound uptake of the ^14^C-ligands, the general protease, “Pronase” (Sigma-Aldrich; Cat. #P8811) was used, implementing established methods [49, 52]. Briefly, cells were treated with ^14^C-chelators (25 μM), washed four times on ice with ice-cold PBS and then incubated on ice with Pronase (1 mg/mL) for 30 min/4°C. Subsequently, cells were removed from the plate on ice and centrifuged at 10,000 rpm/1 min/4°C. The supernatant obtained represents the Pronase-sensitive membrane-bound fraction and the pellet was resuspended in PBS and represents the Pronase-insensitive internalized compartment. Radioactivity in each fraction was assessed as described above.

### ^14^C-Dp44mT Cellular Efflux

Examination of ^14^C-Dp44mT release from pre-labeled SK-N-MC cells was performed using standard techniques [34, 35]. Briefly, SK-N-MC cells were incubated in a similar manner to uptake studies and were pre-labeled with ^14^C-Dp44mT (25 μM) in the presence or absence of HSA (40 mg/mL) for 120 min/37°C. The cells were then placed on ice, the media removed and the cell monolayer washed four times with ice-cold PBS. Phenol red-free media containing HSA (40 mg/mL; 1 mL; 37°C) was then added to each plate and the cells were re-incubated at 37°C for up to 60 min/37°C. At the end of each reincubation period, the cells were placed on ice and the overlying media was placed into scintillation vials to estimate the level of extracellular ^14^C-chelator. Then PBS (1 mL) was added to the cells, which were subsequently removed from the plates using a plastic spatula. This suspension was placed into β-scintillation vials and represents cellular-associated ^14^C-chelator. Radioactivity was determined using a β-counter. Results were expressed as % of ^14^C-chelator released into the medium.

### Western Blot Analysis

Protein extraction from cells and western blot analysis were performed using established protocols [90]. The primary antibodies used were rabbit anti-human calreticulin antibody (1:500; Cat. #2891, Cell Signaling, Boston, MA, USA), goat anti-human cubilin antibody (1:1000; Cat. #sc-23644, Santa Cruz, CA, USA), rabbit anti-human neonatal Fc receptor (FcRn) antibody (1:500; Cat. #sc-66892, Santa Cruz), mouse anti-human heterogeneous nuclear ribonucleoprotein (hnRNP) A2/B1 antibody (1:1000; Cat. #9304, Cell Signaling), rabbit anti-human secreted protein acidic and rich in cysteine (SPARC) antibody (1:1000; Cat. #5240, Cell Signaling), rabbit anti-human cleaved poly ADP-ribose polymerase (PARP) antibody (1:1,000; Cat. #9541S, Cell Signaling) and mouse anti-human β-actin antibody (1:10,000; Cat. #A5316, Sigma-Aldrich). β-actin was used as a loading control. Enhanced Chemiluminescence (ECL) Plus™ Western Blotting Detection Reagent (GE Healthcare, Australia) was used for detection and images were processed using the ChemiDoc™ MP Imaging System (Bio-Rad, Hercules, CA). Densitometric analysis of western blots was performed using Quantity One software (Bio-Rad, Hercules, CA) and normalized implementing the relative β-actin loading control.

### Labeling of Human Tf with ^59^Fe and ^125^I and Human Serum Albumin with ^125^I

Human Tf (Sigma-Aldrich) was labeled with ^125^I and ^59^Fe (Perkin Elmer Life and Analytical Sciences) using standard methods [50, 51]. HSA was labeled with ^125^I using the chloramine-T method [91]. Non-protein bound ^125^I was removed by chromatography using PD10 desalting columns (VWR International, Australia). Further desalting was conducted using Millipore Amicon Ultra-15 Ultrafiltration device (>30 kDa; Billerica, MA). Trichloroacetic acid (Sigma-Aldrich) precipitation was used to determine labeling and desalting efficiency, which was >95%. Protein concentration was measured using UV-visible spectrophotometer (UV-1800; Shimadzu, Kyoto, Japan) at 279 nm (ε_HSA_ = 0.531 g/L; [5]). Competition studies of the ^125^I-HSA with non-labeled HSA demonstrated the labeled protein retained its conformation.

### ^125^I-HSA Cellular Uptake

Cellular uptake of ^125^I-HSA was performed as described above in *“Cellular Uptake of^14^ C-Ligands”* with modifications. SK-N-MC cells were incubated with ^125^I-HSA (0.001-10 mg/mL) in FCS-free media in the presence and absence of unlabeled Dp44mT (25 μM) for 2 h/37°C. In subsequent studies performed as a function of time, cells were incubated with ^125^I-HSA at a concentration of 7.5 mg/mL, as it provided appropriate levels of cellular labeling. The uptake of ^125^I-HSA was examined in the presence and absence of unlabeled Dp44mT, Bp4eT or PIH (25 μM) over 5-30 min/37°C. At the end of the incubation, cells were placed on ice, the medium removed and cells washed four times with ice-cold PBS. Cells were then incubated with Pronase (1 mg/mL) for 30 min/4°C to separate the membrane-bound and internalized ^125^I-HSA fractions. Radioactivity was measured on a γ-counter (Wallac Wizard 3, Perkin Elmer Life and Analytical Sciences).

### Non-Linear Regression Analysis of Ligand Binding to Cells

Non-linear regression analysis was performed in studies examining cellular ^14^C-ligand or ^125^I-labeled HSA uptake as a function of concentration using GraphPad Prism 5.0 Software (San Diego, CA) to measure the maximum number of binding sites (*B_max_*) and the equilibrium dissociation constant (*K_d_*).

### Effect of Metabolic and Endocytosis Inhibitors, Temperature, Lysosomotropic Agents and Excess Ligand on the Cellular Uptake of ^14^C-Dp44mT, ^125^I-HSA and ^59^Fe-^125^I-Tf

The cellular uptake of ^14^C-Dp44mT, ^125^I-HSA or ^59^Fe-^125^I-Tf in the presence or absence of metabolic and endocytosis inhibitors, lysosomotropic agents or at 4°C, was performed as described above in the *“Cellular Uptake of^14^ C-Ligands”* and “*^125^ I-HSA Cellular Uptake*” sections with modifications. SK-N-MC cells were pre-incubated for 30 min/37°C with: (1) FCS-free media; (2) FCS- and glucose (Glu)-free media (−Glu); (3) FCS- and Glu-free media containing the known metabolic inhibitors (Sigma-Aldrich), sodium azide (NaN_3_; 30 mM) or sodium cyanide (NaCN; 5 mM) used previously [34, 35]; (4) FCS-free media at 4°C; (5) FCS-free media containing the well characterized endocytosis inhibitor, phenylglyoxal (PGO; 5 mM; Sigma-Aldrich) [64-67]; or (6) FCS-free media containing the lysosomotropic agents (Sigma-Aldrich), ammonium chloride (NH_4_Cl; 15 mM) or methylamine (MeNH_2_; 15 mM) [52, 65, 68]; or (7) a 100-fold excess of unlabeled Fe-Tf or HSA (75 μM) at 37°C unless otherwise stated.

The media were then removed and the cells subsequently incubated under the seven preincubation conditions listed above in the presence of ^14^C-Dp44mT (25 μM) with or without HSA (40 mg/mL), ^125^I-HSA (0.75 μM) or ^59^Fe-^125^I-Tf (0.75 μM) for 1 h at 37°C unless otherwise stated. Radioactivity in the ^14^C-Dp44mT-containing samples was then measured using the β-counter above. The intracellular levels of ^125^I-HSA, ^59^Fe or ^125^I-Tf were examined by measuring the radioactivity of the Pronase-insensitive (internalized) fraction using the γ-counter, as described above.

### Cell Growth Inhibition Assay by Direct Cell Counts

SK-N-MC cells were seeded in 24-well plates at a density of 7.5 × 10^4^ cells/well in 500 μL of complete culture medium and incubated overnight at 37°C. After this incubation, cells were treated for 24 h/37°C with Dp44mT (30, 60 or 120 μM), Bp4eT (30, 60 or 120 μM), PIH (125, 250 or 500 μM) or the vehicle alone (control) in complete MEM containing diferric Tf (1.2 μM; Sigma-Aldrich) in the presence or absence of HSA (40 mg/mL). Cells were then harvested using PBS/EDTA, resuspended and stained using Trypan blue (0.4%; Sigma-Aldrich). Cell counts and viability were assessed by direct manual counting using a hemocytometer. Results were expressed as a percentage of the control and the concentration required to inhibit growth by 50% (IC_50_) was calculated.

### Statistical Analysis

Data was expressed as mean ± S.E.M of at least 3 experiments. Statistical analysis was performed using Student's *t*-test.

## SUPPLEMENTARY MATERIAL AND FIGURES


