# Supplementary material for: Potentiating the cellular targeting and anti-tumor activity of Dp44mT via binding to human serum albumin: two saturable mechanisms of Dp44mT uptake by cells
Source: Oncotarget. 2015 Mar 15;6(12):10374–98. doi: 10.18632/oncotarget.3606 (PMC4496362; doi:10.18632/oncotarget.3606)
Supplement: Supplementary file 1 [file oncotarget-06-10374-s001.pdf]

Potentiating the cellular targeting and anti-tumor activity of Dp44mT *via* binding to human serum albumin: two saturable mechanisms of Dp44mT uptake by cells

Supplementary Material

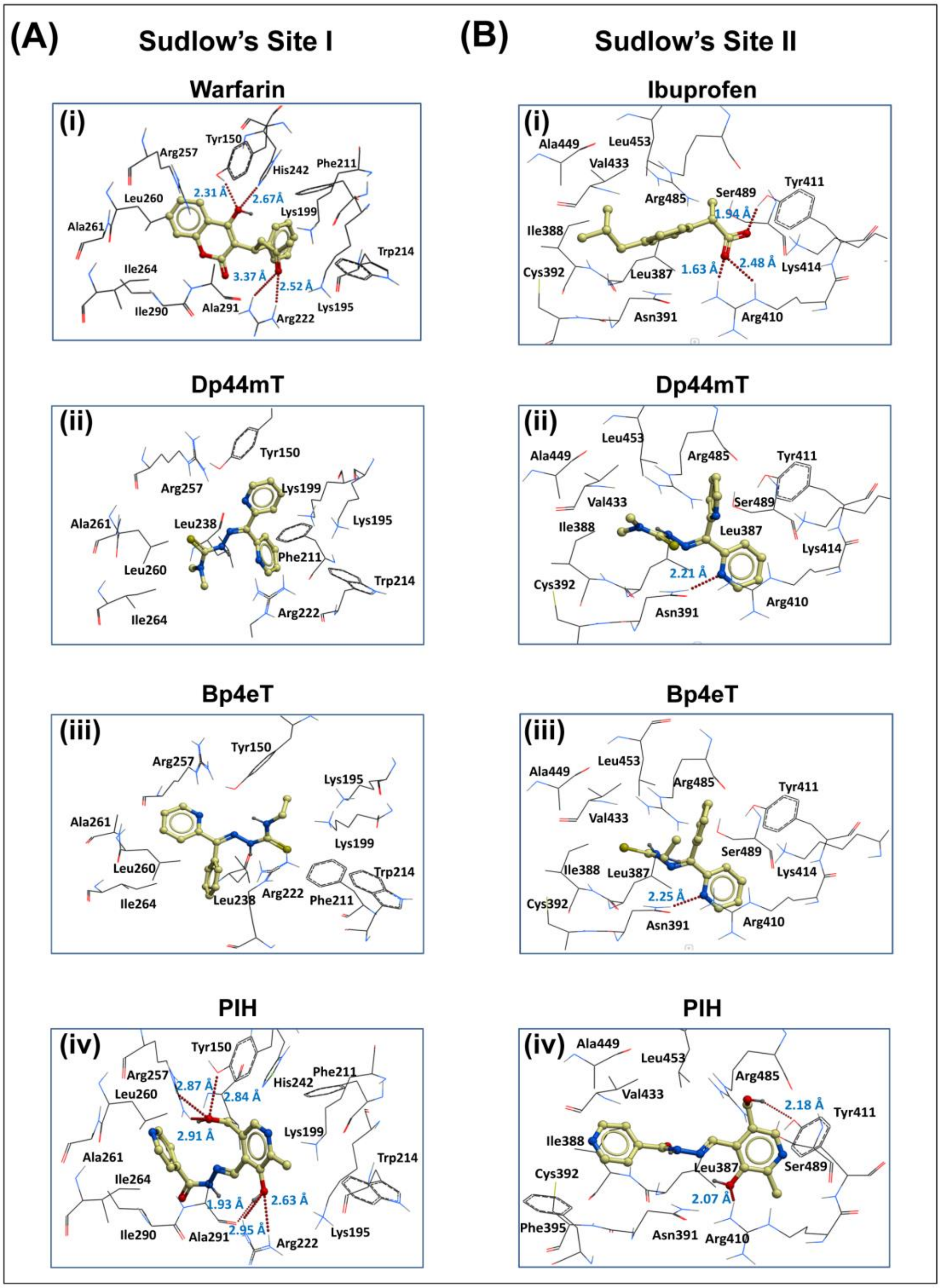

Supplementary Figure 1: Docking results of warfarin, ibuprofen, Dp44mT, Bp4eT and PIH to HSA in: (A) Sudlow's site I (sub-domain IIA) and (B) Sudlow's site II (sub-domain IIIA). (A) No direct H-bonds were formed between Dp44mT and Bp4eT, and Sudlow's site I, while PIH formed H-bonds with Tyr150, Arg422, Arg257 and Ala291 at this site. (**B**) At Sudlow's site II, Dp44mT and Bp4eT made a H-bond with Asn391, while PIH made H-bonds with Arg410 and Tyr411. H-bond distances are given in Å units.

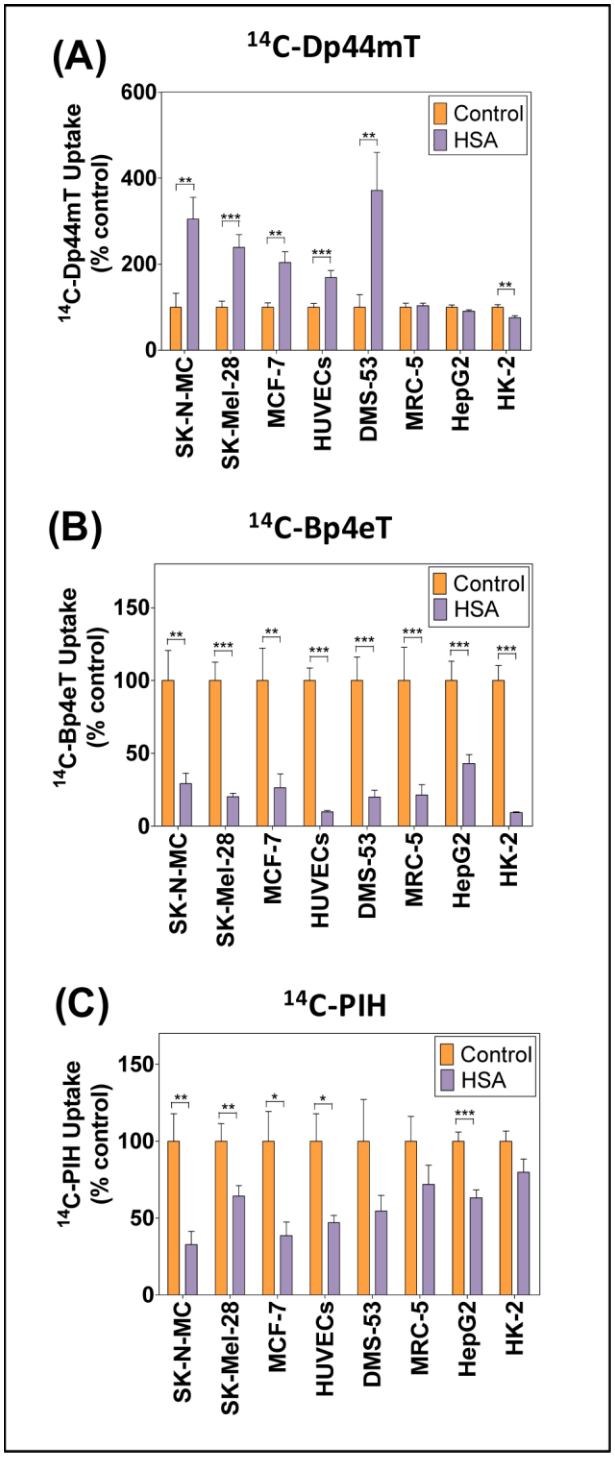

Supplementary Figure 2: Effect of HSA on the uptake of: (A) ^{14}C-Dp44mT, (B) ^{14}C-Bp4eT and (C) ^{14}C-PIH in a variety of cell lines at 37°C. Immortal cell-types (*i.e.,* SK-N-MC, SK-Mel-28, MCF-7, DMS-53, HepG2 and HK-2) and mortal cells (*i.e.,* MRC-5 and HUVECs) were

incubated with 25 μM of ^{14}C-chelator in HSA-containing (40 mg/mL) media or protein-free media for 2 h/37°C. The cells were then washed 4 times using ice-cold PBS and the radioactivity was quantified. Results are expressed as mean ± S.E.M. of at least 3 experiments. *, $p<0.05$; **, $p<0.01$; ***, $p<0.001$ relative to the control.
